# LONGL-Net: temporal correlation structure guided deep learning model to predict longitudinal age-related macular degeneration severity

**DOI:** 10.1093/pnasnexus/pgab003

**Published:** 2022-03-19

**Authors:** Alireza Ganjdanesh, Jipeng Zhang, Emily Y Chew, Ying Ding, Heng Huang, Wei Chen

**Affiliations:** Department of Electrical and Computer Engineering, Swanson School of Engineering, University of Pittsburgh, Pittsburgh, PA 15261, USA; Department of Biostatistics, Graduate School of Public Health, University of Pittsburgh, Pittsburgh, PA 15213, USA; Division of Epidemiology and Clinical Applications, National Eye Institute, National Institutes of Health, Bethesda, MD 20892, USA; Department of Biostatistics, Graduate School of Public Health, University of Pittsburgh, Pittsburgh, PA 15213, USA; Department of Electrical and Computer Engineering, Swanson School of Engineering, University of Pittsburgh, Pittsburgh, PA 15261, USA; Department of Biostatistics, Graduate School of Public Health, University of Pittsburgh, Pittsburgh, PA 15213, USA; Division of Pulmonary Medicine, Department of Pediatrics, UPMC Children's Hospital of Pittsburgh, University of Pittsburgh, Pittsburgh, PA 15219, USA

**Keywords:** age-related macular degeneration, deep learning, Generative Adversarial Networks, longitudinal outcome prediction

## Abstract

Age-related macular degeneration (AMD) is the principal cause of blindness in developed countries, and its prevalence will increase to 288 million people in 2040. Therefore, automated grading and prediction methods can be highly beneficial for recognizing susceptible subjects to late-AMD and enabling clinicians to start preventive actions for them. Clinically, AMD severity is quantified by Color Fundus Photographs (CFP) of the retina, and many machine-learning-based methods are proposed for grading AMD severity. However, few models were developed to predict the longitudinal progression status, i.e. predicting future late-AMD risk based on the current CFP, which is more clinically interesting. In this paper, we propose a new deep-learning-based classification model (LONGL-Net) that can simultaneously grade the current CFP and predict the longitudinal outcome, i.e. whether the subject will be in late-AMD in the future time-point. We design a new temporal-correlation-structure-guided Generative Adversarial Network model that learns the interrelations of temporal changes in CFPs in consecutive time-points and provides interpretability for the classifier's decisions by forecasting AMD symptoms in the future CFPs. We used about 30,000 CFP images from 4,628 participants in the Age-Related Eye Disease Study. Our classifier showed average 0.905 (95% CI: 0.886–0.922) AUC and 0.762 (95% CI: 0.733–0.792) accuracy on the 3-class classification problem of simultaneously grading current time-point's AMD condition and predicting late AMD progression of subjects in the future time-point. We further validated our model on the UK Biobank dataset, where our model showed average 0.905 accuracy and 0.797 sensitivity in grading 300 CFP images.

Significance Statement We develop a framework for automated grading and future prediction for age-related macular degeneration (AMD) severity based on current visit's Color Fundus Photograph (CFP). We aim to answer 2 main questions in clinical practice regarding AMD severity that are “what is the current condition of a subject?” and “how will their condition change until their next visit?” simultaneously in our classification model. We also develop a Generative Adversarial Network model capable of predicting next visits’ CFPs based on the one for the current visit, which can provide interpretability for the classifier's predictions. Our framework can get readily used for other diseases as well, provided that they have several stages and their main diagnostic modality be image data.

## Introduction

Age-related macular degeneration (AMD) is a neurodegenerative irreversible disease that leads to gradual vision loss due to dysfunction of the central retina and its supporting elements (1, 2). AMD is the leading cause of blindness in developed countries and is responsible for 9% of visual loss in the world. (3, 4) Both environmental and genetic factors have been shown to affect the AMD progression (5–12), and 288 million people are estimated to develop AMD globally in 2040 (13), which will impose a large burden on eye service providers. It is both time-consuming and expensive for image specialists or physicians to grade images for the diagnostic purpose. Therefore, developing automated grading and predictive models for facilitating AMD diagnosis has great potential in clinic practice, particularly in the low-resource areas.

AMD severity grading is primarily done by examining color fundus photographs (CFP) by ophthalmologists. A total of 3 stages are defined for AMD: early, intermediate, and late (advanced). Early and intermediated AMD includes the presence of drusen of varying size and most patients are asymptomatic. Severe visual loss happens in the late AMD stage that appears in 2 forms: (1) choroidal neovascularization (NV or “Wet” AMD), in which leaky blood vessels growth harms the outer retina and photoreceptor cells that leads to visual loss (14, 15). (2) Geographic atrophy (GA or “dry” AMD) includes the loss of retinal pigment epithelium (RPE) and photoreceptors, and hence, decrease in sensitivity of the retina to light stimuli (16, 17). Late AMD may develop as one or a combination of these 2 late forms.

Deep-learning (DL)-based methods have achieved promising results in medical research, and they have been leveraged in a wide range of modalities such as AMD severity grading, Alzheimer disease degeneration prediction using neuroimaging data, longitudinal studies with MRI scans for behavioral predictions, cancer outcome prediction, diagnosis of neurological emergencies, drug discovery, cell type identification, and so on (18–26). Their success in automatic medical image classification and segmentation tasks mainly relies on 2 aspects: (1) Convolutional Neural Network (CNN) architectures for feature extraction. In traditional machine-learning algorithms, the input features to the classifier were usually determined by domain experts such as clinicians or by heuristic methods. In contrast to traditional machine-learning algorithms that the input features to the classifier are usually determined by domain experts (e.g. clinicians) or heuristic methods, CNN includes a stack of convolution layers as its feature extractor followed by a classifier where the weights of the convolution layers and the classifier are “learned” during training by adjusting the weights to minimizing a loss function. In other words, CNN learns useful features for its classification task from the training data, and it is not forced to use some predetermined handmade features for classification. (2) Availability of large-scale datasets provided by long-term studies such as Age-Related Eye Disease Study (AREDS) and UK Biobank (UKB) image datasets (16, 27–29). These datasets with accurate clinical labels enable CNN to achieve high accuracy after extensive training.

In recent years, several papers have proposed DL methods using color fundus images for prediction. Some have focused on developing a model that determines the AMD status (w/o late AMD), AMD severity score, probability of GA, or probability of neovascular AMD development at each time-point (visit) based on its fundus images (17, 18, 30–33). These methods can provide an accurate grading tool. Nevertheless, they are not capable of predicting progression to advanced AMD, which is more clinically desirable because it enables clinicians to start preventive actions such as prescribing AREDS nutritional supplements to make the progression of vulnerable subjects to late AMD slow, considering that late AMD is currently incurable and irreversible. The recent 2 studies (15, 34) have used fundus images w/o AMD-associated independent genetic variants of the subjects to predict whether the AMD progression time for them will be longer than a predetermined duration or not (2–7 years in (15) and 1–5 years in (34)). Bridge et al. (35), inspired by advances in sequence modeling architectures (36, 37), have developed a model which uses a sequence of longitudinal images to predict the AMD risk of the subject in the future. The main challenge for using sequence models, especially Recurrent Neural Networks (RNN) such as Long Short-Term Memory (LSTM) (37) and Gated Recurrent Units (GRU) (36), in longitudinal studies for future prediction is that these models assume that their input is in a time-series format where the gaps between their successive inputs in different sample sequences are equal such as in sentences. However, it is common in longitudinal datasets to have heterogeneity in the gaps between consecutive visits of participants and the patterns of visit times of different participants, which makes the longitudinal datasets different from the time-series data. Bridge et al. (35) have proposed interval scaling to alleviate this problem. Nonetheless, their method needs at least 2 or 3 previous time-point images to predict the condition in future time-points with high performance that is not feasible for the first-time visiting subjects.

In this paper, we propose a new DL-based model, LONGL-Net, to tackle the challenges of both the paradigms described above and unify their strengths. At first, we train a classification model to simultaneously predict (1) current time-point's AMD condition (binary diagnosis: advanced or not advanced) as well as (2) the subject's disease condition (advanced or not advanced) in the future (given an inquired time-point; e.g. 3 years later) based on the fundus image at the current time. These 2 questions are the most important clinical questions during the patient's visit after collecting the fundus image. To address these 2 prediction tasks, we develop a new temporal-correlation-structure guided Generative Adversarial Network (GAN) model capable of learning the correlation and pattern of AMD progression between the prospectively collected images at each study intervals having distinct AMD trajectories in the training data. This model can examine the current time-point's fundus image and perform longitudinal predictions for the future time-points’ fundus images based on it. The predicted images can provide visualization interpretability for the classification model's decision for ophthalmologists by predicting how important signs of AMD such as drusen size or pigmentary abnormalities severity of the subjects will be in the future time-points. We should note that the difference between our model and the one proposed in (15) is twofold: (1) Our model learns the temporal interrelations between the images at baseline and future time-point, and utilizes these knowledge to enhance the longitudinal outcome prediction. As a result, our model can predict the fundus image at future time-point ((15) cannot do that) to support the decisions and make them interpretable. (2) Our model's prediction contains more clinical outcomes as it predicts the AMD status of both the current time-point and the future one (i.e. the longitudinal outcome). In other words, the problem that we address is simultaneously grading the current time-point's image and predicting the future status, which is different from (15) that only performs the latter. Our experiments demonstrate the effectiveness of the classifier and GAN model both quantitatively and qualitatively.

## Methods

To predict a subject's AMD condition in the future time-point, we propose a classification model that takes the current time-point's fundus image as input and generates outputs: (1) current time-point's AMD severity condition as well as (2) a predicted AMD condition for the given future time-point. We design a new GAN model, which learns the interrelations of temporal changes between the images at successive time-points, and by doing so, it will be competent to predict the future time-point's eye fundus image. This generated image can get examined by a physician to prescribe necessary preventive actions and make the model interpretable for ophthalmologists. We elaborate details of our model in the following subsections.

### Problem formulation

We denote the time gap between 2 visits (current time-point and the future one) in a pair in the unit of 6 months with $T$. For example, $T\ = \ 4$ means 2 years gap, and we assume that $T$ is chosen beforehand. For the ${i{\mathrm{ th}}}$ participant, we represent the pair for time-points ${t_j}\ $and ${t_{j + 1}}$ with ${X_{ij}} = \ ( {{x_{i,\ {t_j}}},\ {x_{i,\ {t_{j + 1}}}}} )$, where ${x_{i,\ {t_j}}}$ is the first time-point's fundus image and ${x_{i,\ {t_{j + 1}}}}$ is the future one's image taken after $T*6$ months. We show the label of the pair $( {{x_{i,\ {t_j}}},\ {x_{i,\ {t_{j + 1}}}}} )$ with ${y_{i,\ j}}$, and define 3 classes: (1) $\ \ {y_{i,\ j}} = \ 0$ when neither ${x_{i,\ {t_j}}}$ nor ${x_{i,\ {t_{j + 1}}}}$ is progressed to advanced AMD. (2) $\ \ {y_{i,\ j}} = \ 1$ if ${x_{i,\ {t_j}}}$ has not progressed to advanced AMD but ${x_{i,\ {t_{j + 1}}}}$ has progressed, and (3) $\ \ {y_{i,\ j}} = \ 2$ if both ${x_{i,\ {t_j}}}$ and ${x_{i,\ {t_{j + 1}}}}$ have progressed to advanced AMD. As advanced AMD is currently irreversible and untreatable, $({x_{i,\ {t_j}}}$, ${x_{i,\ {t_{j + 1}}}}):\ ( {Adv,\ not\ Adv} )$ is not possible yet. The goal is to use the first time-point available in each pair $({x_{i,\ {t_j}}})$ to (1) predict the label ${y_{i,\ j}}$, which according to the encoding system that we used, determines AMD condition of the current time-point and predicts the future time-point's severity, and (2) estimate how the fundus image of the future time-point ${x_{i,\ {t_{j + 1}}}}$ will look like by generating $x_{i,\ {t_{j + 1}}}^{\prime}$ and training the model in such a way to make $x_{i,\ {t_{j + 1}}}^{\prime}\ $and ${x_{i,\ {t_{j + 1}}}}$ as close as possible.

### Late AMD progression prediction

We train a model to not only classify the current time-point's AMD disease condition, but also predict the one for the future time-point. By doing so, we can avoid the challenge of sequence models to handle time-series data with uneven gaps, because let us assume there is a subject with available visit times {1, 4, 5, 8}. In this case, the sequence model should handle a heterogeneous sequence with the gaps (3, 1, 3), but in our approach, we can train a model to predict the condition of 4 time-points (2 years) later readily by using the pairs {(1, 5), (4, 8)}.

We train the classification model using the “Weighted Cross Entropy” loss function so that the model takes the imbalance of the dataset into account by penalizing wrong predictions that the model performs in the minority classes more than the ones in majority classes. We use the histogram of the training set to calculate the class weights as follows:

\begin{eqnarray*} &&Training\ Set\ Histogram:h\ = \ \left[ {{h_1},{h_2},\ \ldots ,\ {h_C}} \right],\ h^{\prime} = \left[ {\frac{1}{{{h_1}}},\ \frac{1}{{{h_2}}},\ \ldots ,\ \frac{1}{{{h_C}}}} \right]\ = \left[ {h_1^{\prime},\ h_2^{\prime},\ \ldots ,\ h_C^{\prime}} \right]\\ &&w\ = \frac{1}{{\mathop \sum \nolimits_{j = 1}^N h_j^{\prime}}}\ \ \left[ {h_1^{\prime},\ h_2^{\prime},\ \ldots ,\ h_C^{\prime}} \right] = \ \left[ {{w_1},\ {w_2},\ \ldots ,\ {w_C}} \right]. \end{eqnarray*}

where $\mathop \sum \limits_{j\ = \ 1}^C {h_j} = \ 1$ and $C$ is the number of classes.

Finally, our classification loss function will be:

\begin{eqnarray*} {\rm{\ }}{{\cal L}_{class}} = \ - \mathop \sum \limits_{i\ = \ 1}^N \mathop \sum \limits_{j\ = \ 1}^{{N_i}} (\mathop \sum \limits_{k\ = \ 1}^C {y_{i,j,k}}{w_k}{\rm{log}}\left( {{p_{\ i,j,k}}} \right)), \end{eqnarray*}

where $N$ is the number of training subjects, ${N_i}$ is the number of pairs available for the subject ${X_i}$, and $C$ is the number of classes which is 3 in our experiments. ${y_{i,\ j,\ k}}$ is the $^{\prime}k^{\prime}$th element of the one-hot encoding vector for the label ${y_{i,\ j}}$ for the pair $({x_{i,\ {t_j}}}$, ${x_{i,\ {t_{j + 1}}}})$, and ${p_{i,\ j,\ k}}$ is the predicted probability of the model for the $^{\prime}k^{\prime}$th class for the input pair $( {{x_{i,\ {t_j}}},\ {x_{i,\ {t_{j + 1}}}}} )$ of the subject ${X_i}$.

### Predicting future time-point's fundus image

DL models are called “black-box” models because the reason behind their predictions is not transparent. We use a GAN model to predict the future time-point's fundus image based on the current one. This prediction can be more useful in clinical settings than saliency maps that highlight parts of the input image that had the most influence on the classifier's decision, because although these methods may provide “where” the network has focused on, they cannot explain “how” these regions will be in the future time-point. Moreover, a model with the ability to predict the future time-point's image enables us to estimate how the fundus image will be in the time-points farther than just one step by repeatedly inputting its prediction in it several times.

#### Temporal correlation structure guided GAN

GAN (38) are the models that are capable of implicitly learning the training data distribution and generating new “realistic looking” data from the learned distribution. Originally, GAN models were designed in a fashion that 2 players called “generator” and “discriminator” compete with each other in a minimax game (38), where the discriminator “*D*” distinguishes whether its input is a “real” data or a “fake” one generated by the generator “*G*”, and the generator attempts to produce realistic-looking samples from an input noise to fool the discriminator. The objective function used for training these 2 networks is as follows:

\begin{eqnarray*} \mathop {\min }\limits_G \mathop {\max }\limits_D {E_{y \sim p\left( y \right)}}\left[ {\log \left( {D\left( y \right)} \right)} \right] + \ {E_{z \sim q\left( z \right)}}[\log \left( {1 - D\left( {G\left( z \right)} \right)} \right]. \end{eqnarray*}

In this objective, $p( y )$ is the unknown real training data distribution that the generator attempts to learn, and $q( z )$ is the input noise distribution that is usually chosen to be Gaussian. “*D*” is the discriminator function that outputs the probability of its input being “real,” and “*G*” is the generator function that maps the input sampled noise into a fake sample. Intuitively, the discriminator aims to output value “1” for the real inputs and “0” for the samples generated by the generator, thereby maximizing the objective. On the other hand, the generator attempts to fool the discriminator to make its output value close to “1” for the generated sample, thereby minimizing the second term, and consequently, the whole objective above. Ideally, as the training continues, the players reach Nash Equilibrium that the generator learns the real data distribution, and the discriminator cannot distinguish between the real and generated inputs.

We frame this problem as predicting the fundus image for a future time-point with a predetermined distance to the current one, using the current fundus image as described in section (problem formulation) There are several characteristics in our problem that the model should consider: (1) as the time distance between the current time-point and the future one is not very large, we can reasonably assume that the general structure of an eye such as the location of the optic disc and major blood vessels in these images should not differ significantly, and only minor details such as drusen size may temporally change. Therefore, we seek that the generator takes the current time-point's fundus image of a subject and generate a sample “conditioned” on it, i.e. consider the input structure in the generation process. (2) In the training, we are not only interested in encouraging the generator to produce a realistic predicted image based on the first (current) time-point input, but also there is a target image (second (future) time-point's fundus image in a training pair) that has certain semantic connections (e.g. AMD features such as larger drusen size) to the input image that we desire our generated image be as close to it as possible concerning semantics. Therefore, we modify the original GAN training procedure to make it suitable for our case. First, the input image is passed to the generator to make generation conditional, and to make it stochastic to cover different modes of the output distribution, dropout (39) is used in the generator architecture (40) instead of inputting noise to the generator. Second, both the first (current) time-point's image and the generated image are passed to the discriminator as a pair so that it can consider whether they have required structural connections. For example, it can recognize that the generated image is not true “pair image” for the first time-point's image, i.e. they are not from the joint distribution of the first and second time-point images $p( {{x_{{t_j}}},\ {x_{{t_{j + 1}}}}} )$ if the optic disc being on the right side of one of them and left of the other. Also, we use a patch-level discriminator for computational efficiency as suggested in the literature (40, 41). Finally, L1 reconstruction loss between the generated and target images is added to the objective as it encourages preserving low-frequency information (40) between the input and output of the generator. Therefore, our GAN model's training objective is as following:

\begin{eqnarray*} &&\mathop {\min }\limits_G \mathop {\max }\limits_D {E_{\left( {{x_{{t_j}}},\ {x_{{t_{j + 1}}}}} \right) \sim p\left( {{x_{{t_j}}},\ {x_{{t_{j + 1}}}}} \right)}}\left[ {\log \left( {D\left( {{x_{{t_j}}},\ {x_{{t_{j + 1}}}}} \right)} \right)} \right]\\ && + \ {E_{{x_{{t_j}}} \sim p\left( {{x_{{t_j}}}} \right)}} [\log \left( {1 - D\left( {{x_{{t_j}}},\ G\left( {{x_{{t_j}}}} \right)} \right)} \right]\\ && + \ {\lambda _r}[{E_{\left( {{x_{{t_j}}},\ {x_{{t_{j + 1}}}}} \right) \sim p\left( {{x_{{t_j}}},\ {x_{{t_{j + 1}}}}} \right)}}\|{x_{{t_{j + 1}}}} - G\left( {{x_{{t_j}}}} \right){\|_1}]. \end{eqnarray*}

**Fig. 1.**
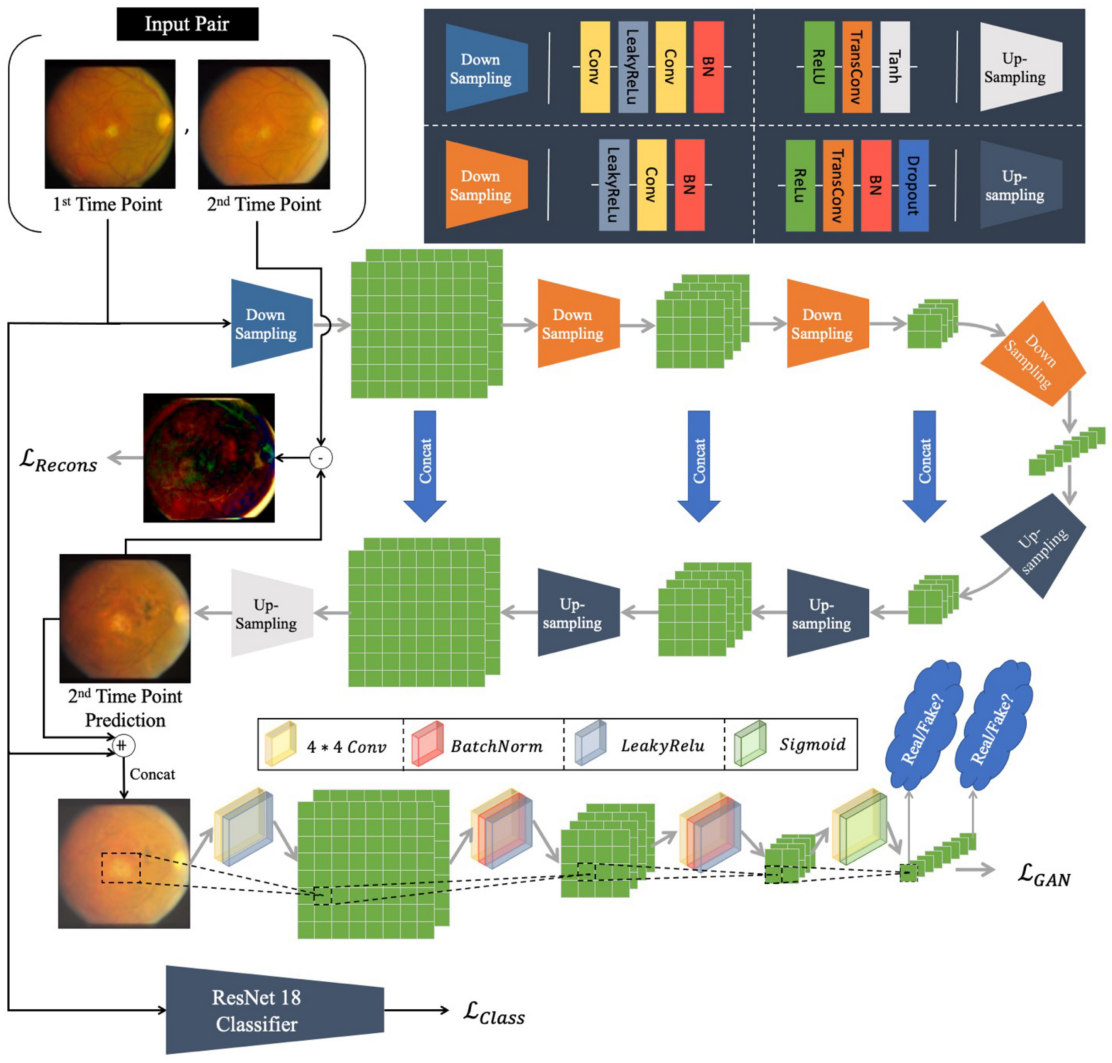
Training procedure and architectures of the classifier and the temporal correlation structure guided GAN model. Given a training input pair, the generator model (U-Net architecture in the middle with shape “$ \supset $”) predicts the fundus image of the second (future) time-point based on the first (current) time-point's image. The L1-norm of the difference of the predicted image and the second time-point (the difference image is magnified for better visualization) in the pair will form the reconstruction loss $( {{{\cal L}_{{\boldsymbol{Recons}}}}} )$. In addition, the first time-point's image and the predicted image get concatenated and passed to the discriminator that evaluates whether the generated image has reasonable structural properties, considering the first time-point's fundus image. Patch-wise discrimination is depicted where the scores on several distinct patches contribute to the final GAN model's loss function. Finally, the classifier is trained by the weighted cross-entropy loss function $( {{{\cal L}_{{\boldsymbol{Class}}}}} )$ using the pair's first time-point's image and its 3-class encoded label.

$p( {{x_{{t_j}}},\ {x_{{t_{j + 1}}}}} )$ is the joint distribution of the first and second time-point images in the training dataset, and ${x_{{t_j}}} \sim p( {{x_{{t_j}}}} )$ is the marginal distribution of the first time-point images. ${\lambda _r}$ is a hyperparameter balancing the generation loss and the reconstruction loss to be tuned. The training procedure as well as the architectures of the proposed classification and GAN models are depicted in Fig. 1.

### Ethics

All the color fundus images and clinical phenotypes from the AREDS dataset are available in dbGaP (accession number phs000001.v3.p1). The UKB dataset were obtained from the UK Biobank Access Management System (application number 43252).

## Experiments

In this section, we describe our experimental settings and the results of our classification and GAN models.

### Data descriptions

We used 2 independent datasets in our experiments: The National Eye Institute (NEI) AREDS (27) and the UKB (29). We used the AREDS dataset for model training, validation, and testing and treated the UKB dataset as an independent validation cohort study to further test our framework.

#### AREDS

The National Eye Institute (NEI) AREDS (phs000001.v3.p1 in dbGaP) is a large-scale, long-term prospective clinical trial of AMD and age-related cataract. A total of 187,996 color fundus images of 4,628 subjects and corresponding eye-level phenotypes are covered in AREDS. In total, 1 subject could have up to 13 years of follow-up visits since the baseline. The stereoscopic fundus images were taken from 30° Zeiss cameras with different stereoscopic fields (28). In this work, we only focused on Field 2 (${30^{\circ}}$ imaging field centered at the fovea), which centered 1/8–1/4 disk diameter above the center of the macula. We selected Field 2 since this angle focuses on the most important region related to AMD (macula) and holds the largest sample size in AREDS. Meanwhile, for most eyes given any visit, both left-side and right-side fundus of the stereoscopic pair images are available for each eye.

We randomly picked all left-side images of the stereoscopic pair of all eyes to avoid redundant information and boost training speed. The overall image quality of fundus images in AREDS is acceptable for the following grading and research. Only 0.6% of images of 48,998 eyes were ungradable through the first 10 years of AREDS (28). The number of images used for training, validation, and testing of different experiments is shown in Table S1 (Supplementary Material S1).

#### UKB

The UKB is a population-scale database comprising genetic and health information of more than half a million participants in the UK (29). A total of 175,546 fundus images of 85,728 subjects from 2 visits (initial visit 2006–2010 and first repeat visit 2012–2013) are available in the UKB database. Corresponding phenotypes were collected by questionnaires, but with large missing values. There are only 1,695 images with definite AMD from questionnaires. Under the guidance of an image specialist, we manually extracted 300 images (200 of controls and 100 with advanced AMD) regarding acceptable image quality and available phenotypes as an independent test set for our experiments.

### Data partitioning

We randomly divide 4,628 participants in the AREDS study into train, validation, and test partitions with the ratio 90% (4,166), 5% (232), and 5% (230), such that all images for each participant lie in one of the partitions. This prevents the possibility of information leakage between 2 different partitions when images of one participant being present in both. We use the mentioned partitioning in all of our experiments below, and the study ID of participants in each partition can be found in our GitHub repository^1^. We tune the model hyperparameters using the validation set and report the performance metrics on the test set.

### Data preprocessing

We followed the same preprocessing protocol in (18), i.e. each color fundus image is cropped to a square which encompasses the macula region, and then, the square image was resized to 224 × 224 pixels. We did not perform other data augmentations such as mirroring, flipping, or rotation used in (32) because our assumption for the GAN model is that the structure of the images such as the position of the optic disc and its relative position to major blood vessels between the current time-point and the future one does not change significantly, but such augmentations can change these structural parameters dramatically, which can mislead the generation process. Some of the original images and their transformed versions can be found in Figures S1–S8 (Supplementary Material S2).

### Experimental settings

We implement all of our models using PyTorch (42) and train them with an NVIDIA TITAN Xp GPU with 12GB memory. We use Adam optimizer (43) in training both the classifier and GAN model.

### Classification model

We train and evaluate the classification model with the pairs with 2, 3, and 4 years gap between the first and second time-points. In contrast with previous works (15, 17, 32–35) that use Inception V3 (44), we use ResNet-18 (45) architecture pretrained on ImageNet (46) as our CNN classifier considering that ResNet architectures are state of the art models for image recognition (45). In each experiment, we perform hyperparameter tuning as follows: for each hyperparameter setting, at first, we train the ResNet-18 model with pretrained weights on ImageNet for 20 epochs and evaluate the model every 50 iterations on the validation set. Finally, we save the checkpoint with the lowest validation loss as the best model of this setting. Among the saved checkpoints for different hyperparameter settings, we select the one with the highest validation AUC as our model. Finally, we calculate the 95% confidence interval for its performance on the validation and test set using the bootstrap method.

We use the 12-level AMD severity-scale labels available in the AREDS dataset for the first and second time-points to define the labels ${y_{i,\ j}}\ $ for each pair. If the AMD severity scale of an eye is within the range from 0 to 9, it is considered as “not progressed to advance AMD,” and if the score is from 10 to 12, the eye is considered as “progressed to advance AMD.” Table 1 summarizes the statistics of the dataset in each setting. As can be seen, the original dataset is extremely imbalanced that most of its available pairs lie in the class ${y_{i,\ j}} = \ 0$, indicating that both the first time-point and the next one have not progressed to advanced AMD. The reason is that most of the eyes of the subjects in the AREDS study were not in advanced AMD condition at the baseline and did not progress until the end of the study (15).

**Table 1.** Original dataset statistics. The numbers in the parenthesis in the columns 4–6 indicate the histogram of each row.

T (time gap in units of 6 months)	Pairs of visits used (if available)	Number of pairs available	Number of pairs ${{\boldsymbol{y}}_{{\boldsymbol{i}},{\boldsymbol{\ j}}}} = {\boldsymbol{\ }}0$	Number of pairs ${{\boldsymbol{y}}_{{\boldsymbol{i}},{\boldsymbol{\ j}}}} = {\boldsymbol{\ }}1$	Number of pairs ${{\boldsymbol{y}}_{{\boldsymbol{i}},{\boldsymbol{\ j}}}} = {\boldsymbol{\ }}2$
4 (2 years)	{(0, 4), . . ., (22, 26)}	50,320	42,205 (83.9%)	1,683 (3.3%)	6,432 (12.8%)
6 (3 years)	{(0, 6), . . ., (19, 26)}	42,862	35,706 (83.3%)	1,999 (4.6%)	5,157 (12.1%)
8 (4 years)	{(0, 8), . . ., (18, 26)}	36,185	29,835 (82.5%)	2,180 (6%)	4,170 (11.5%)

High imbalance in the dataset will make training the classifier challenging, because if the classifier predicts all the samples belonging to the class with major samples $({y_{i,\ j}} = \ 0)$ and neglect minority classes $({y_{i,\ j}} = \ 1)$, it can still achieve high accuracy and low loss value even if we use the weighted cross-entropy loss function. Therefore, we down-sample the available pairs for each subject with the following procedure:

For each eye:

1. If a participant has no pairs with ${y_{i,\ j}} = \ 0$, we use all of their pairs with ${y_{i,\ j}} \ne 0$.
2. If a participant has no pairs with ${y_{i,\ j}} \ne 0$, we randomly choose one pair with ${y_{i,\ j}} = \ 0$.
3. If a participant has $n > 0$ pairs with ${y_{i,\ j}} \ne 0$ and $m > 0$ pairs with ${y_{i,\ j}} = \ 0$:
4. If $n < m$, we use all $n$ nonzero pairs as well as randomly selected $n$ pairs with ${y_{i,{\rm{\ }}j}} = {\rm{\ }}0$.
5. If $n \ge m$, we use all $n$ nonzero pairs as well as all $m$ pairs with the label zero.

After applying the procedure above, the number of pairs for each setting (in total and each of train/validation/test partitions) is shown in Table S1 (Supplementary Material S1). We utilize Scikit-learn (47) implementation of the generalization of AUC score for multiclass classification problems introduced by Hand and Till (48) and calculate the confidence intervals using 2000 bootstrap samples. For each of the partitions, if we denote the size of it with “N,” we sample “N” samples with replacement from it for each bootstrap iteration.

### Temporal correlation structure guided GAN model

Similar to the classification model, we train the GAN model for 2, 3, and 4 years future prediction. U-Net 256 architecture (49) and 70 × 70 patch discriminator (40, 41) are used as our generator and discriminator, respectively. The batch-size is set to 1 to prevent the generated samples from getting correlated as a result of the combination of their statistics in the batch normalization layers (50). The network is trained for 200 epochs. Adam optimizer's parameters are set to $( {learning\ rate:0.0002,\ {\beta _1}:0.5,\ {\beta _2}:0.999} )$. The learning rate is constant for the first 100 epochs and then is linearly decayed to 0 for the next 100 epochs. More details about the GAN model architecture are available in Supplementary S3.

To evaluate the GAN model, at first, we train a model for each gap value and show some longitudinal predictions made by them to assess the visual quality of the generated images. Generally, there is no principled approach to quantitively evaluate the quality of the generated images (40). Here, we explore 4 experimental settings to get some sense of how good the generated images are. The high-level idea is inserting the GAN-generated images into a classifier and checking the prediction performance of their sequential combination compared to other baselines.

### Qualitative evaluation

Our GAN model can be clinically acceptable if it can correctly predict the changes in AMD characteristics such as drusen size or pigmentary abnormalities, and we explore these characteristics in predicted images as a qualitative evaluation of generated images. We leverage the images for subjects whom we have their {baseline 4th, 6th, and 8th} time-point images and use the baseline one to make a longitudinal prediction for the others and compare them with their ground truth images in terms of AMD-related features. Predicting the transition from not advanced to advanced AMD is the most difficult case for the model because (1) each GAN model does not have access to any label or information about the next time-point's condition during generation. They are trained using the time-points with a certain gap (2, 3, or 4 years) and deployed for generation using only the baseline test image. (2) The dataset is imbalanced, such that most of the pairs are both in not advanced AMD or both are in advanced and the progressing pairs are the minority, and the GAN model learns more about the first 2 categories. Hence, we examine the GAN model's predictions for a “transitioning” subject here.

### Quantitative evaluation

We design 4 experiments and compare their results to check the quantitative performance of our GAN model as follows. In all cases, the goal is to predict whether the second (future) time-point in a given pair is in advanced AMD condition or not.

### Predicting second time-point's AMD condition using the GAN model and 3-class classifier sequentially

**Fig. 2.**
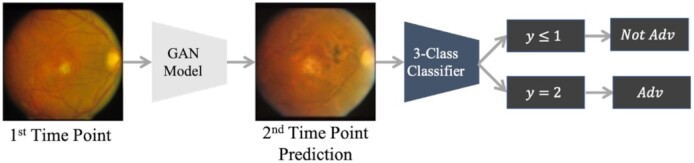
The procedure of predicting the second time-point's AMD condition using the GAN model and the classifier sequentially. The GAN model predicts the second time-point's image, and the classifier predicts a label ${\boldsymbol{^{\prime}y^{\prime}}}$ which will be decoded as Not Adv AMD or Adv AMD in the second time-point.

Figure 2 illustrates our first experiment's scheme. We leverage the GAN model to predict the second time-point's fundus image based on the first one in a pair. Then, we ask our trained classification model to perform a prediction about the AMD condition of the generated image. As described in section (problem formulation), the classifier predicts 1 of 3 possible classes $y\ \epsilon \ \{ {0,\ 1,\ 2} \}$, where $y\ \epsilon \ \{ {0,\ 1} \}$ represents the cases that the input image is not in advanced AMD condition. Therefore, we map the predicted labels of the classifier to binary ones in terms of its prediction for the generated image's AMD condition, i.e. $y\ \epsilon \ \{ {0,\ 1} \}$ gets mapped to “0” because in these cases, the input image is predicted to not be in advanced AMD and $y\ = \ 2$ to “1” vice versa.

We name this model ‘GAN + 3C-Classier’ in our comparisons. For the classifiers, we use the trained checkpoints of the models described in the section (Classification Model).

### Predicting the next time-point's AMD condition using the GAN model and binary classifier

**Fig. 3.**
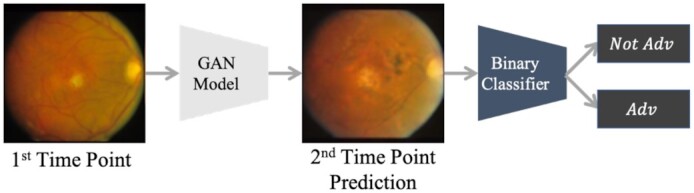
The procedure of predicting the second time-point's AMD condition using the GAN model and a binary classifier. The GAN model's prediction is used by the binary classifier to predict second time-point's label.

As another experiment, first, we train a binary classifier that predicts whether its input is in the advanced AMD condition or not. Then, similar to the previous part, we use the sequential connection of the GAN model and the binary classifier to predict the AMD condition of the second time-point using the first time-point's image. The procedure is shown in Fig. 3.

We use the same data partitioning—in terms of study subjects—that we used for our multiclass classifiers to train the binary classifier. The only difference is that we utilize both samples and their binary labels in the pairs for training. The trained binary classifier's performance is 0.9708 AUC and 0.9488 accuracy on the validation set, and 0.976 as well as 0.949 on the test set. We call this model ‘GAN + B-Classifier.’

### Predicting next time-point's AMD condition using our trained multiclass classifier

As some baselines to compare the performance of the models in the sections (Predicting Second Time Point’s AMD Condition Using the GAN Model and 3-class Classifier Sequentially) and (Predicting the Next Time Point’s AMD Condition Using the GAN Model and Binary Classifier) with, we check the performance of our multiclass classifier to predict the second time-point's AMD condition in 2 settings: (1) Using the first time-point's image: in our coding system for the labels of the multiclass classifier, $y\ \epsilon \ \{ {1,\ 2} \}$ indicates that the second time-point will be in advanced AMD. Therefore, we decode the predicted labels of the classifier to “not Adv” when the predicted label is zero and “Adv” otherwise. We show this case with “3C-Classifier, First Time point.” (2) Using the second time-point's image: the second time-point in the pair is the input of the classifier. Now, the input is the image that we are going to predict its label. Therefore, if the classifier output label is $y\ \epsilon \ \{ {0,\ 1} \}$, it means that the prediction is for the second time-point is “not Adv” and “Adv” otherwise. We represent this case with “3C-Classifier, Second Time Point.”

### Validations of the model using independent UKB dataset

To examine the generalizability of our model, we evaluate it on the independent UKB dataset. In general, the quality of the fundus images in UKB is inferior to the ones in AREDS, and a part of the dataset is completely ungradable because of severe issues with images. Further, the main difference between AREDS and UKB is in the labeling scheme where AREDS labels are provided by image reading center (28), but the labels in UKB are gathered by collecting the participants’ answers to questionnaires regarding their AMD condition. In AREDS, a 12-level severity score of AMD condition of the subjects is available. However, the labels in UKB are the self-reported status of the subjects to the questions asking whether a doctor has diagnosed them with AMD (yes/no).

Due to the differences between the datasets, it is not feasible to directly evaluate the performance of our classifier in the same manner as before, i.e. checking its predictions about the current AMD state of a subject and its condition in the next time-point. At first, the dataset contains records of 3 visits (indexed with 0, 1, and 2) of the participants, and the age of subjects at the first visit (visit 0) is available, but the age of only a limited part of subjects is available at visits 1 and 2, which is essential for our model to determine the gap between visit pairs. Secondly, the labels in the UKB dataset can only indicate whether a subject has been diagnosed with AMD, but they do not reveal whether the subject has progressed to advanced AMD or not, and the subjects with AMD can be in the early or intermediate stages of the disease. Therefore, we evaluate our model on the task of only grading the first time-point images, i.e. labeling whether the input image is in advanced AMD condition or not (binary classification).

Although the labels for “adv”/’not adv’ AMD condition is not available, an image specialist helped us to pick 100 images with severe drusen size and pigmentary abnormality condition as an approximate advanced AMD test set and 200 images with the label “not diagnosed with AMD” from the dataset as a control set. All the selected images are chosen such that at least the macula region of the image is visible and not defected due to imaging procedure issues. The name of images for both advanced AMD and the control group are listed in Supplementary (Images from UK Biobank Used in Our Experiments), (Supplementary Material S6).

## Results

### Late AMD progression prediction

Table 2 demonstrates the prediction accuracy, AUC, and confusion matrix of our classifier for different time-gap values. All models show high AUC (∼90%) and accuracy (∼75%), which is desirable in unbalanced problems where AUC is a more reliable metric than accuracy. From confusion matrices, we can calculate that all models can grade current time-point images that are not in advanced AMD (class “0” and “1”) with an accuracy better than 90%, and also, the ones that are in advanced AMD now (the class “2”) with more than 80% accuracy. Furthermore, all models have about 70% accuracy in correctly predicting the cases that are not in advanced AMD now but will progress to it in the next time-point (class “1”) that are the most challenging cases because they are the minority class in the dataset, and the classifier can observe fewer examples from them compared to other classes during training. High performance of all models suggests the capability of CNN to accurately perform diagnosis for the current time-point as well as predict AMD condition of the next one.

**Table 2.** Performance of the classifier for different time gaps. The numbers in parenthesis indicate 95% confidence intervals which have been calculated by the bootstrap method. The best hyperparameter settings for each model are described in Table S2 (Supplementary Material S4).

T (time gap in units of 6 months)	Validation accuracy	Validation AUC	Test accuracy	Test AUC
4 (2 years)	0.752 (0.7238, 0.779)	0.8832 (0.8629, 0.9026)	0.7715 (0.7455, 0.7975)	0.896 (0.875, 0.9146)
4, Confusion matrix	\begin{eqnarray*} [{\begin{array}{@{}*{3}{c}@{}} {327}&{114}&7\\ {24}&{62}&{10}\\ {10}&{55}&{278} \end{array}} ] \end{eqnarray*}		\begin{eqnarray*} [{\begin{array}{@{}*{3}{c}@{}} {329}&{100}&{10}\\ {16}&{58}&{10}\\ {12}&{54}&{295} \end{array}} ] \end{eqnarray*}	
6 (3 years)	0.7356 (0.7062, 0.765)	0.9059 (0.888, 0.9213)	0.7465 (0.7143, 0.7773)	0.8988 (0.8795, 0.9176)
6, Confusion matrix	\begin{eqnarray*} [{\begin{array}{@{}*{3}{c}@{}} {290}&{125}&{10}\\ {19}&{78}&{20}\\ 4&{38}&{233} \end{array}} ] \end{eqnarray*}		\begin{eqnarray*} [ {\begin{array}{@{}*{3}{c}@{}} {274}&{103}&9\\ {15}&{74}&{19}\\ 3&{48}&{232} \end{array}} ] \end{eqnarray*}	
8 (4 years)	0.7724 (0.7416, 0.8032)	0.9199 (0.905, 0.9337)	0.7688 (0.7382, 0.8008)	0.9209 (0.9050, 0.9353)
8, Confusion matrix	\begin{eqnarray*} [ {\begin{array}{@{}*{3}{c}@{}} {283}&{93}&{10}\\ {25}&{96}&{16}\\ 3&{20}&{198} \end{array}} ] \end{eqnarray*}		\begin{eqnarray*} [ {\begin{array}{@{}*{3}{c}@{}} {257}&{91}&{14}\\ {14}&{82}&{21}\\ 2&{24}&{213} \end{array}} ] \end{eqnarray*}	

### Model's decision interpretation using saliency maps

**Fig. 4.**
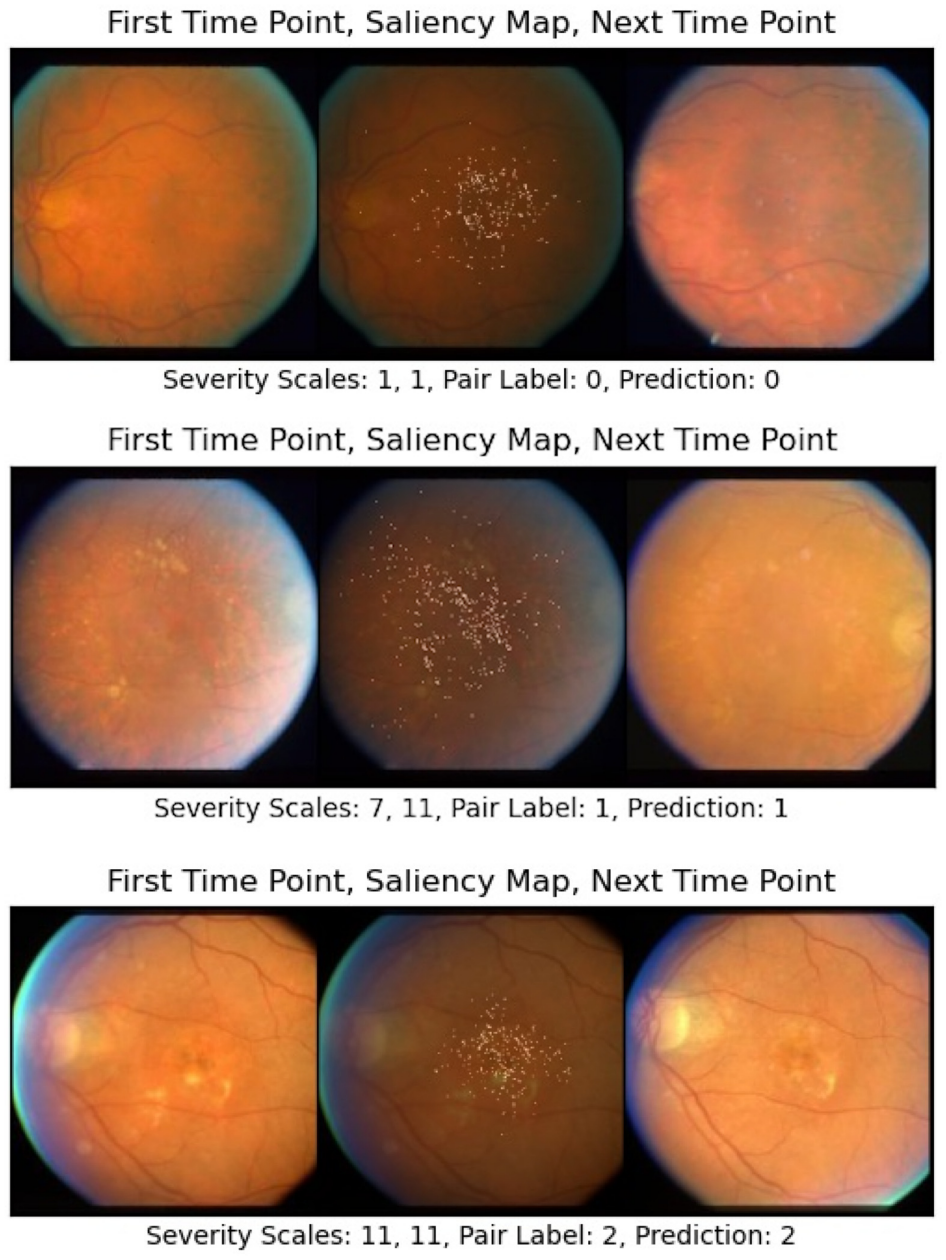
Each row from left to right: first time-point's image, saliency map of it, and second time-point's image. All images are chosen from the test set. The first time-points’ images’ names are “52,340_04_F2_LE_LS,” “52,759_06_F2_RE_LS,” and “3923_08_LE_F2_LS,” respectively. As can be seen in the saliency maps, the classifier has focused on the macula region and its properties such as drusen size and pigmentary abnormalities, which is aligned with the clinical process of AMD diagnosis.

To obtain intuitions about how the model has reached its decision, we visualize saliency maps of the input images that highlight the regions of the fundus images that had the most influence on the classifier's outcome. Generally, physicians grade the AMD score of a fundus image based on the properties of their macula region. Therefore, the decisions of the classifier will be more convincing if we observe that it has focused on these areas as well. Figure 4 illustrates 3 pairs (each row represents a pair) in the test set from 3 possible classes, i.e. the first time-point (left image) and the second one (right image) have AMD conditions (not adv, not adv), (not adv, adv), and (adv, adv), respectively. The images in the middle exhibit the saliency maps over the first time-points’ fundus images. We can observe that the points in the macula had the most impact on the classifier's decision, which is aligned with the clinical approach for grading fundus images.

### Using age phenotype in addition to fundus images

As the name of AMD suggests, age is an important factor contributing to AMD. Therefore, we explored whether giving the age information explicitly to the network will boost the results or not. To do so, we concatenated the age value to the output of the global average pooling layer of the ResNet classifier, and the resulting vector is passed to the fully connected classifier. The whole scheme is shown in Figure S14 (Supplementary Material S8). We did not find a significant difference in the accuracy and AUC values (less than 1% in all cases) of the classifier on the validation and test sets, which has been also reported in (15). We conjecture that the reason may be that the effect of aging is comprehensible for the classifier from the fundus images, and inputting age information does not provide additional information for the model.

### Predicting next time-point's fundus image

#### Qualitative results

**Fig. 5.**
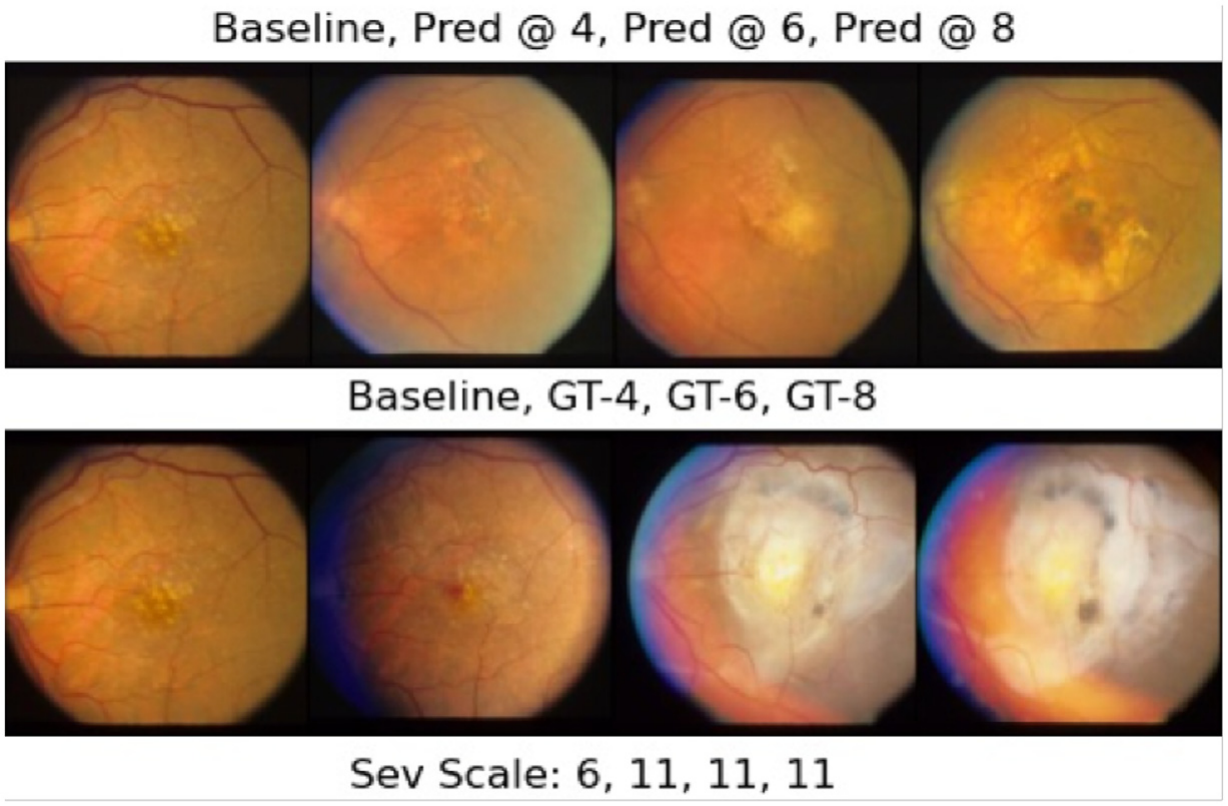
Longitudinal prediction for the left eye of a subject who has progressed to advanced AMD. Top row from left to right: baseline, prediction at 4th, 6th, and 8th time-points (2, 3, and 4 years, respectively) after baseline. Bottom row from left to right: baseline, ground truth (GT) images for the 4th, 6th, and 8th time-points after baseline. The images in each column are the prediction and their corresponding ground truth. The AMD severity scale of each ground truth image is shown in the bottom line. The name of the baseline image in the AREDS dataset is “51,662_QUA_F2_LE_LS.jpg.”

**Fig. 6.**
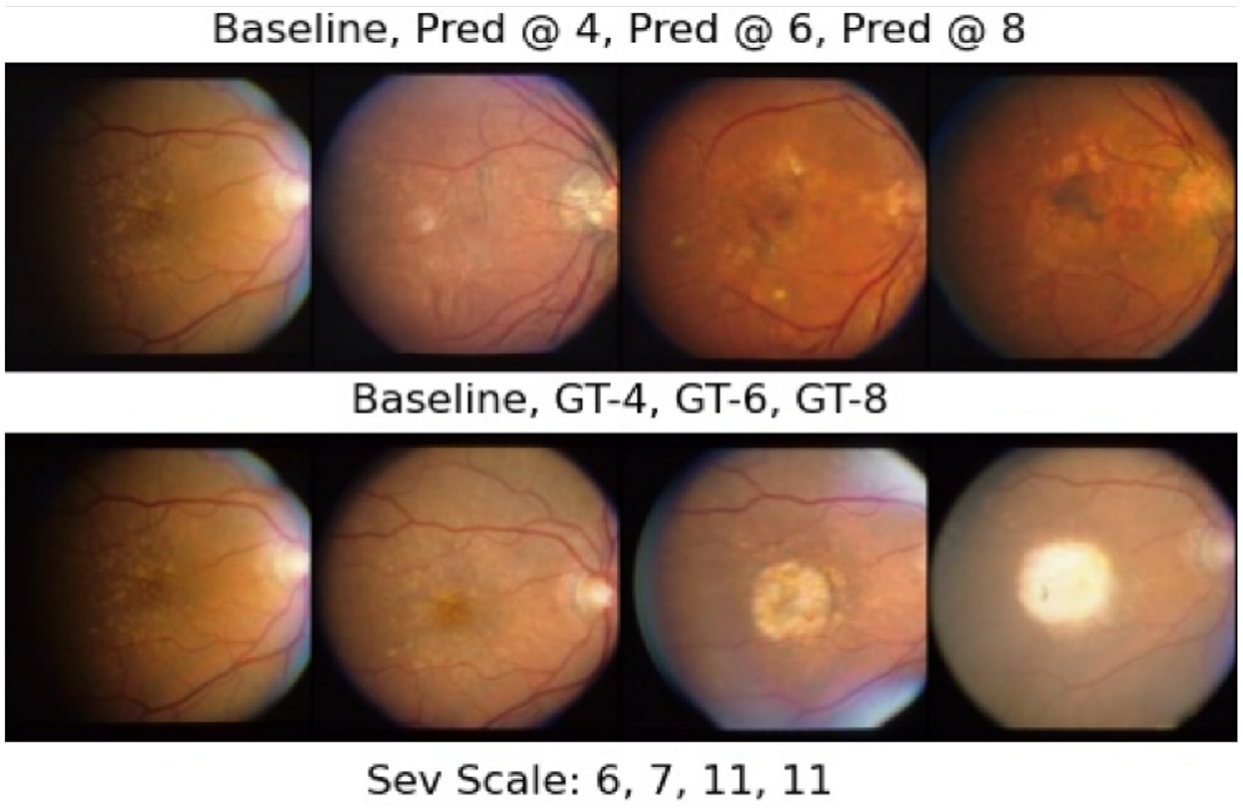
Longitudinal prediction for the right eye of a subject who has progressed to advanced AMD. Top row from left to right: baseline, prediction at 4th, 6th, and 8th time-points after baseline. Bottom row from left to right: baseline, ground truth (GT) images for the 4th, 6th, and 8th time-points after baseline. The images in each column are the prediction and their corresponding ground truth. The AMD severity scale of each ground truth image is shown in the bottom line. The name of the baseline image in the AREDS dataset is “51,662_QUA_F2_RE_LS.jpg.”

Figures 5 and 6 demonstrate our GAN model's longitudinal prediction (first rows) for each eye of a subject in the test set whose both eyes have progressed from not advanced AMD in the baseline to advanced AMD during the study. As can be seen in the ground truth images in the figures (second rows), both eyes experienced enlarging drusen in the macula region from left to right, which has attributed to larger AMD severity scores. We can observe that our model's predictions have the following properties: (1) first and foremost, in Fig. 5, the drusen size of the predictions grow in consecutive time-points (yellow areas in the middle). In Fig. 6, the larger drusen along with some pigmentary abnormalities (black parts) in the macula are predicted. In both cases, the predictions are compatible with the ground truth images’ trajectory from the clinical perspective that cares about the drusen size and the macular region. (2) Secondly, we can observe in Fig. 6 that the left side of the baseline fundus image (the first row, left image) in the dataset is lost, but interestingly, the GAN model can generate some realistic-looking “more complete” image based on the baseline in its prediction for the 4th time-point, which indicates that the model has learned the underlying distribution of the consecutive time-point images properly. We provide more examples of our longitudinal predictions for the other AMD progression cases (not advanced to not advanced, and both being advanced) in Figures S9–S13 (Supplementary Material S5).

#### Quantitative comparison

Table 3 summarizes the results for the 4 cases described for quantitative evaluation of the GAN model. As can be seen, the sequential models for (GAN model → multiclass classifier) have about 70% accuracy, and the ones for (GAN model → binary classifier) perform better (∼75%). The reason may be that multiclass classifier is trained to predict both the first time-point's condition and the second one's, and the number of its training examples for the task of “binary classification of a single time-point” is lower than the binary classifier. In addition, the multiclass classifier using the first time-point performs slightly worse than the one which uses the second time-point. This is expectable because the goal is to predic the second time-point's label, and doing so is easier using the second time-point's image than using the first one.

**Table 3.** Comparison of the GAN model's performance with the baselines.

Model	T (time gap in units of 6 months)	Validation accuracy	Test accuracy
GAN + 3C-Classifier	4 (2 years)	0.743	0.7127
	6 (3 years)	0.7491	0.7181
	8 (4 years)	0.6707	0.6671
GAN + B-Classifier	4 (2 years)	0.7554	0.7342
	6 (3 years)	0.7503	0.7529
	8 (4 years)	0.7376	0.766
3C-Classifier, first time-point	4 (2 years)	0.8253	0.8439
	6 (3 years)	0.8066	0.8327
	8 (4 years)	0.8206	0.8315
3C-Classifier, second time-point	4 (2 years)	0.8873	0.8733
	6 (3 years)	0.8715	0.8636
	8 (4 years)	0.8407	0.8315

Overall, the models containing GAN models have about 70% accuracy that indicates that the GAN models are learning something meaningful between the consecutive time-points which will be useful for prediction by the classifier.

### Validation results on UKB dataset

In the label encoding scheme described in the methods section, labels “0” and “1” predicted by the classifier indicates that the input image is not in advanced AMD condition and “2” means they are progressed to advanced AMD, and we convert the predicted labels accordingly to a binary label. Table S3 (Supplementary Material S7) summarizes the accuracy and confusion matrix of the models for 2(A), 3(B), and 4(C) year gaps in the grading current time-point's image task. We can observe that all models achieve high accuracy on the validation task (∼86%, 92%, and 92%, respectively). Models B and C have high sensitivity as well (86% and 90%), but model A shows relatively lower sensitivity (63%), which suggests that we should rely on models B and C if we are interested in grading the current time-point images.

## Discussion

We proposed a unified framework using advanced DL methods for grading the current time-point's image as well as predicting the future AMD image and condition given a future time-point. By doing so, we aimed to have a one-stop solution that overcomes the limitation of previous methods. Some methods suffer from not providing AMD condition in the future, and some need at least 2 or 3 not advanced AMD prior time-points’ images (which are often not available at practical clinical setting) to perform a good prediction about the future, but our model only needs the current time-point image.

In addition, we proposed a temporal correlation structure guided GAN model, which can learn the dynamics and interrelations of the temporal transition between consecutive time-points properly and perform longitudinal prediction for the next time-points’ images, thereby providing interpretability for the classifier's decision in the clinical setting where a specialist can get more intuition about the major symptoms of the subject in the next time-points based on the predicted images. Through extensive experiments, we evaluated the performance of our trained GAN model, where for qualitative results we case studied a subject who gradually progressed to advanced AMD, and the model could successfully predict enlargement of drusen as well as the progressive appearance of pigmentary abnormalities in the eyes of the subject. For quantitative results, we showed that the sequential connected model consisting of the GAN model and the classifier can achieve about 70% accuracy in predicting the second time-point's AMD condition based on the first one's in the pairs which demonstrate that the GAN model can successfully learn the process of conversion between successive time-points’ images.

As our problem formulation unifies both grading current time-point's CFP and predicting AMD status of the future time-point, a direct performance comparison between our model and the previous prediction models such as (15) is not possible. Therefore, we compare our model's performance with DeepSeeNet (33), which is a grading model. In brief, DeepSeeNet trains 3 modules to grade drusen size, pigmentary abnormalities, and whether the input image is in late AMD or not, and it combines the predictions of these 3 models to predict AMD simplified severity score. Here, we explore our model's performance on the task of grading its input image in comparison with the DeepSeeNet model's late AMD prediction module. The input to our model is the first time-point available in each pair, and according to our label coding, $y\ \epsilon \ \{ {0,\ 1} \}$ indicates that the first time-point is not in advanced AMD and vice versa. Therefore, we combine these 2 classes in the confusion matrix of our model's predictions in Table 2 to obtain a binarized confusion matrix in Table S4 (Supplementary Material S9). As can be seen in Table 4 in DeepSeeNet paper (33), their late AMD grading model has an overall accuracy of 0.967, which is higher than the one for our models. (0.902, 0.898, and 0.915) However, our models show significantly higher values for sensitivity (0.817, 0.819, and 0.891) compared to 0.627 while maintaining close specificity performance (0.962, 0.943, and 0.926) compared to 0.987, which is more useful in practice because false positives are less detrimental than false negatives in medical research.

Our proposed method has some limitations. At first, we mainly focused on the AREDS dataset, the only large-scale longitudinal AMD study available, in our training and evaluations in which most of the subjects have not been in advanced AMD at the baseline and have not progressed to late AMD during the study. This brings about the class imbalance in the training of a classifier which may bias the classifier toward the majority classes. Even though we leveraged the UKB dataset to validate the classifier, further evaluations on other cohorts may be beneficial. Moreover, in this study, we only considered fundus images similar to ophthalmologists for AMD grading, but it has been shown that AMD is associated with genetics, and further incorporating genetics information can improve the prediction performance. In addition, we did not explicitly consider the possible correlation between the AMD condition of the right and left eyes of a subject in our predictions.

A challenge in our GAN model is the fact that the fundus images in the consecutive time-points in the AREDS dataset are not registered, which can mislead the GAN model's training in that it may result in large values for the reconstruction loss, although the predicted image is similar to the ground truth. Also, in some pairs, the major blood vessels or optic disc may be less visible in one of the images compared to the other one due to lightning conditions of the imaging environment or problems in the image acquisition devices, which can similarly lead to suboptimal network training.

In conclusion, our study indicated that automatic grading and prediction methods for AMD disease can achieve reasonable performance. In our future work, we plan to develop a multimodal learning approach to leverage both fundus images and genetic information of the subjects to predict their AMD severity. Besides, leveraging registration methods to register consecutive time-points’ fundus images can greatly benefit the training procedure of the GAN model, which can result in image predictions by the GAN model with higher quality and more accurate details. Our method will likely facilitate the diagnosis and prediction of AMD and other retinal diseases toward precision medicine.

## Supplementary Material

pgab003_Supplemental_File

## Data Availability

The AREDS dataset is available at Database of Genotypes and Phenotypes (dbGaP): https://www.ncbi.nlm.nih.gov/projects/gap/cgi-bin/study.cgi?study_id = phs000001.v3.p1. The UK Biobank (UKB) dataset is accessible through UK Biobank resources: https://biobank.ndph.ox.ac.uk/crystal/. All of our implementations and trained checkpoint models can be found in our GitHub repository https://github.com/Alii-Ganjj/LongitudinalAMDNet.

## Footnotes

1
https://github.com/Alii-Ganjj/LongitudinalAMDNet
