# Supplementary material for: LONGL-Net: temporal correlation structure guided deep learning model to predict longitudinal age-related macular degeneration severity
Source: PNAS Nexus. 2022 Mar 19;1(1):pgab003. doi: 10.1093/pnasnexus/pgab003 (PMC8962776; doi:10.1093/pnasnexus/pgab003)
Supplement: pgab003_Supplemental_File [file pgab003_supplemental_file.pdf]

Supplementary Materials for LONGL-Net: Temporal Correlation Structure Guided Deep Learning Model to Predict Longitudinal Age-related Macular Degeneration Severity

Alireza Ganjdanesh[1,*], Jipeng Zhang[2,*], Emily Y Chew[3], Ying Ding[2], Heng Huang[1,$] , Wei Chen[2,4,$]

[1]Department of Electrical and Computer Engineering, Swanson School of Engineering, University of Pittsburgh, Pittsburgh, PA.
[2]Department of Biostatistics, Graduate School of Public Health, University of Pittsburgh, Pittsburgh, PA.
[3]Division of Epidemiology and Clinical Applications, National Eye Institute, National Institutes of Health, Bethesda, MD.
[4]Division of Pulmonary Medicine, Department of Pediatrics, UPMC Children's Hospital of Pittsburgh, University of Pittsburgh, Pittsburgh, PA.

[*] equal contribution

[$] To whom correspondence should be addressed.
Email: heng.huang@pitt.edu; wec47@pitt.edu

S1. Dataset Statistics after Down-sampling

Table S1 summarizes the number of pairs with 2, 3, and 4 years gap between the first time point and the future one used in training/validation/test sets in our experiments for the classifier and GAN model. In each experiment, the data partitions are disjoint in subject level, i.e., the pairs for each participant are only present in one of data partitions.

Table S1: Statistics of the dataset used for training the classifier after down-sampling. The numbers in the parenthesis in columns 4-6 indicate the histogram of the partition in each row. As the maximum follow-up visit length is 13 years, the largest possible value in the visit pairs is 26.

T (Time Gap in Units of 6 Months), Partition	Pairs of visits used (if available)	Number of pairs available	Number of pairs $\boldsymbol{y_{i,j}} = \mathbf{0}$	Number of pairs $\boldsymbol{y_{i,j}} = \mathbf{1}$	Number of pairs $\boldsymbol{y_{i,j}} = \mathbf{2}$
4, Total	{(0, 4), …, (22, 26)}	16545	8430 (50.9%)	1683 (10.2%)	6432 (38.9%)
4, Train	{(0, 4), …, (22, 26)}	14774	7543 (51%)	1503 (10.2%)	5728 (38.8%)
4, Validation	{(0, 4), …, (22, 26)}	887	448 (50.5%)	96 (10.8%)	343 (38.7%)
4, Test	{(0, 4), …, (22, 26)}	884	439 (49.7%)	84 (9.5%)	361 (40.8%)
6, Total	{(0, 6), …, (20, 26)}	14951	7795 (52.1%)	1999 (13.4%)	5157 (34.5%)
6, Train	{(0, 6), …, (20, 26)}	13357	6984 (52.3%)	1774 (13.3%)	4599 (34.4%)
6, Validation	{(0, 6), …, (20, 26)}	817	425 (52%)	117 (14.3%)	275 (33.7%)
6, Test	{(0, 6), …, (20, 26)}	777	386 (49.7%)	108 (13.9%)	283 (36.4%)
8, Total	{(0, 8), …, (18, 26)}	13654	7304 (53.5%)	2180 (16%)	4170 (30.5%)
8, Train	{(0, 8), …, (18, 26)}	12189	6553 (53.8%)	1926 (15.8%)	3710 (30.4%)
8, Validation	{(0, 8), …, (18, 26)}	747	389 (52%)	137 (18.4%)	221 (29.6%)
8, Test	{(0, 8), …, (18, 26)}	718	362 (50.4%)	117 (16.3%)	239 (33.3%)

S2. Pre-processed Data Examples

The following figures illustrate some original images from batches 2010 and 2014 in the dataset and their corresponding cropped and resized version used in our experiments.

Batch 2010:

1. Image Name: '1638_04_LE_F2_LS'

   Original:

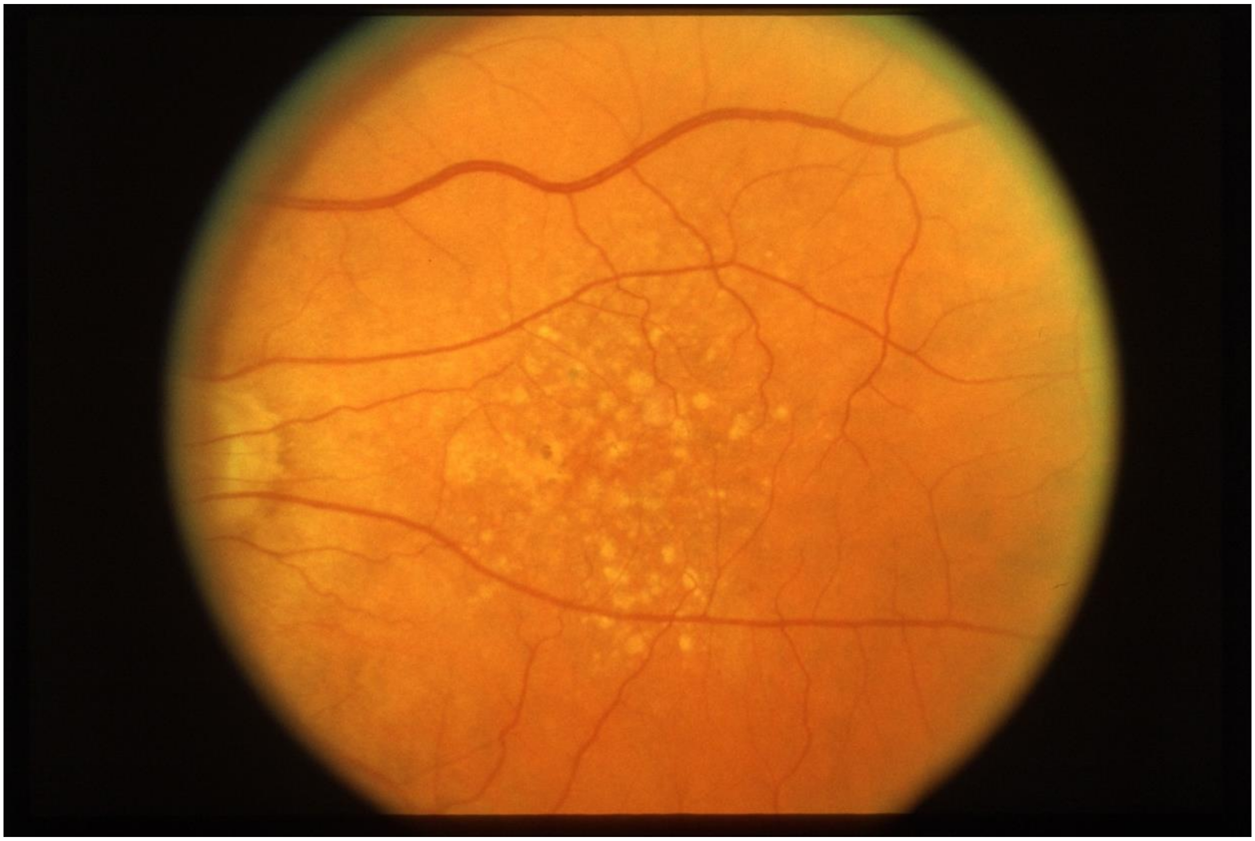

Figure S1: A fundus image from AREDS dataset.

Preprocessed:

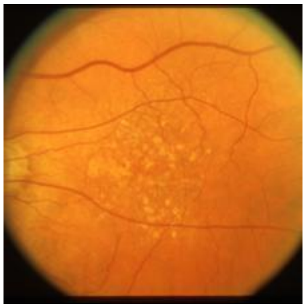

Figure S2: A preprocessed fundus image from AREDS dataset.

2. Image Name: ‘5653_16_LE_F2_LS’

Original:

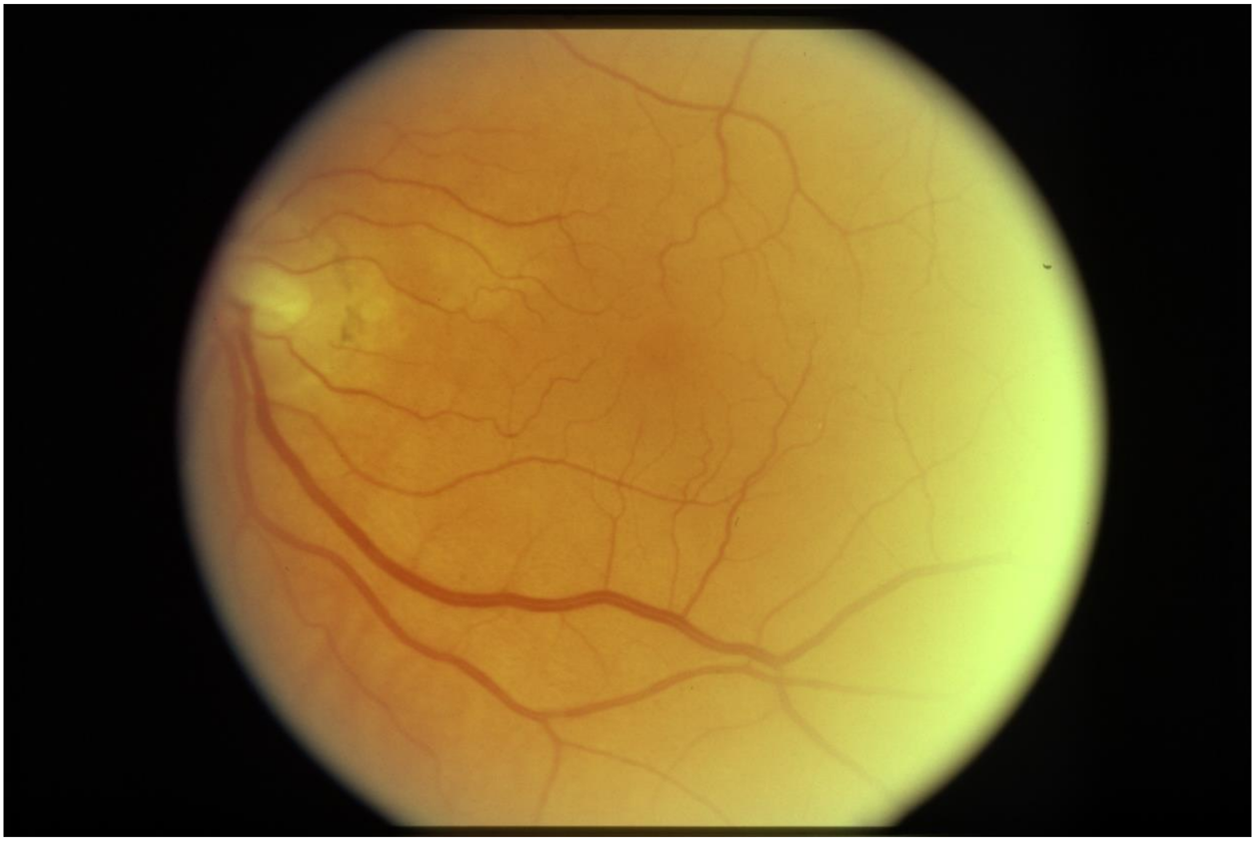

Figure S3: A fundus image from AREDS dataset.

Preprocessed:

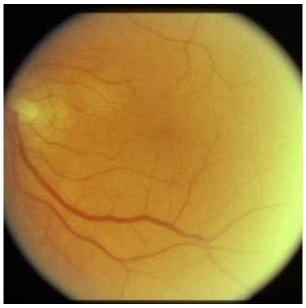

Figure S4: A preprocessed fundus image from AREDS dataset.

Batch 2014:

1. Image Name: ‘52705_QUA_F2_LE_LS’

   Original:

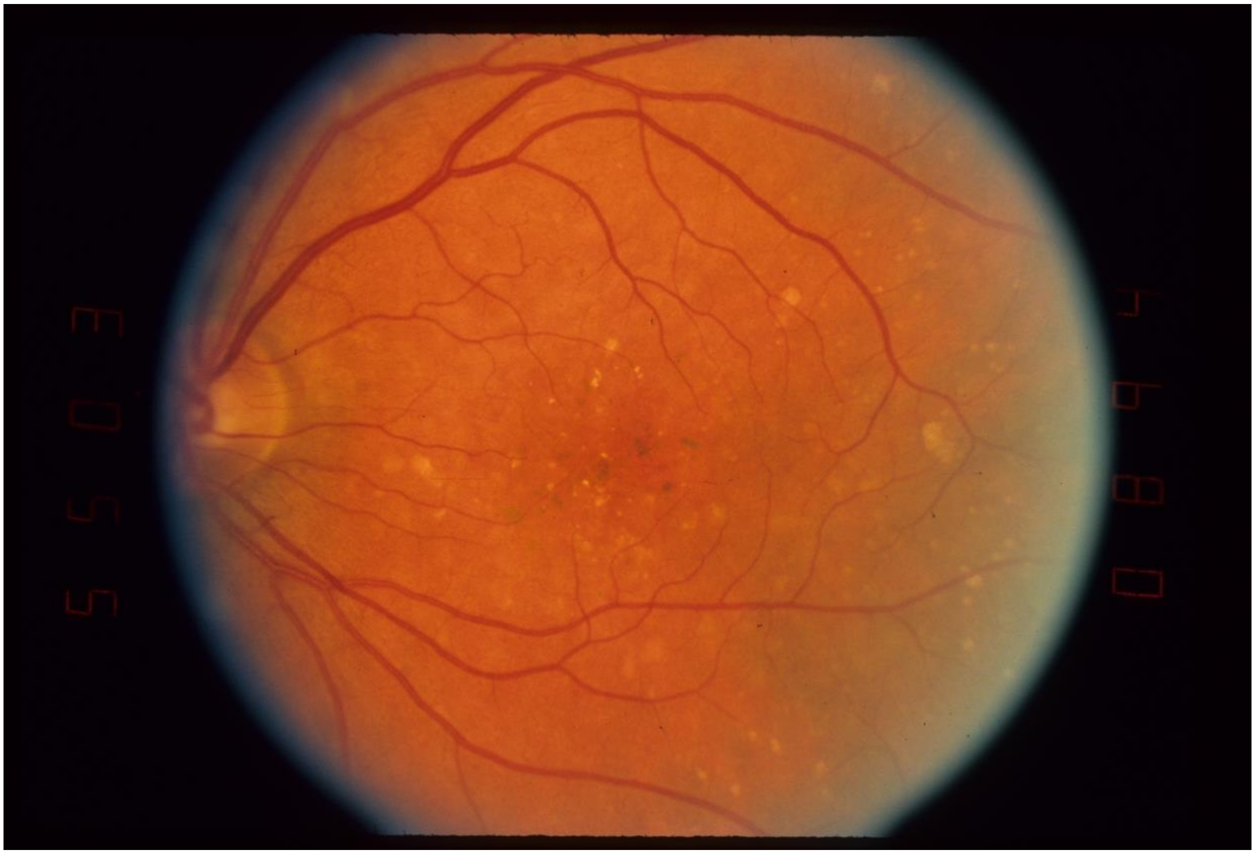

Figure S5: A fundus image from AREDS dataset.

Preprocessed:

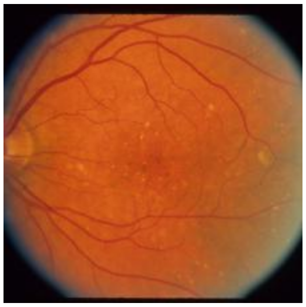

Figure S6: A preprocessed fundus image from AREDS dataset.

2. Image Name: '52634_QUA_F2_LE_LS'

Original:

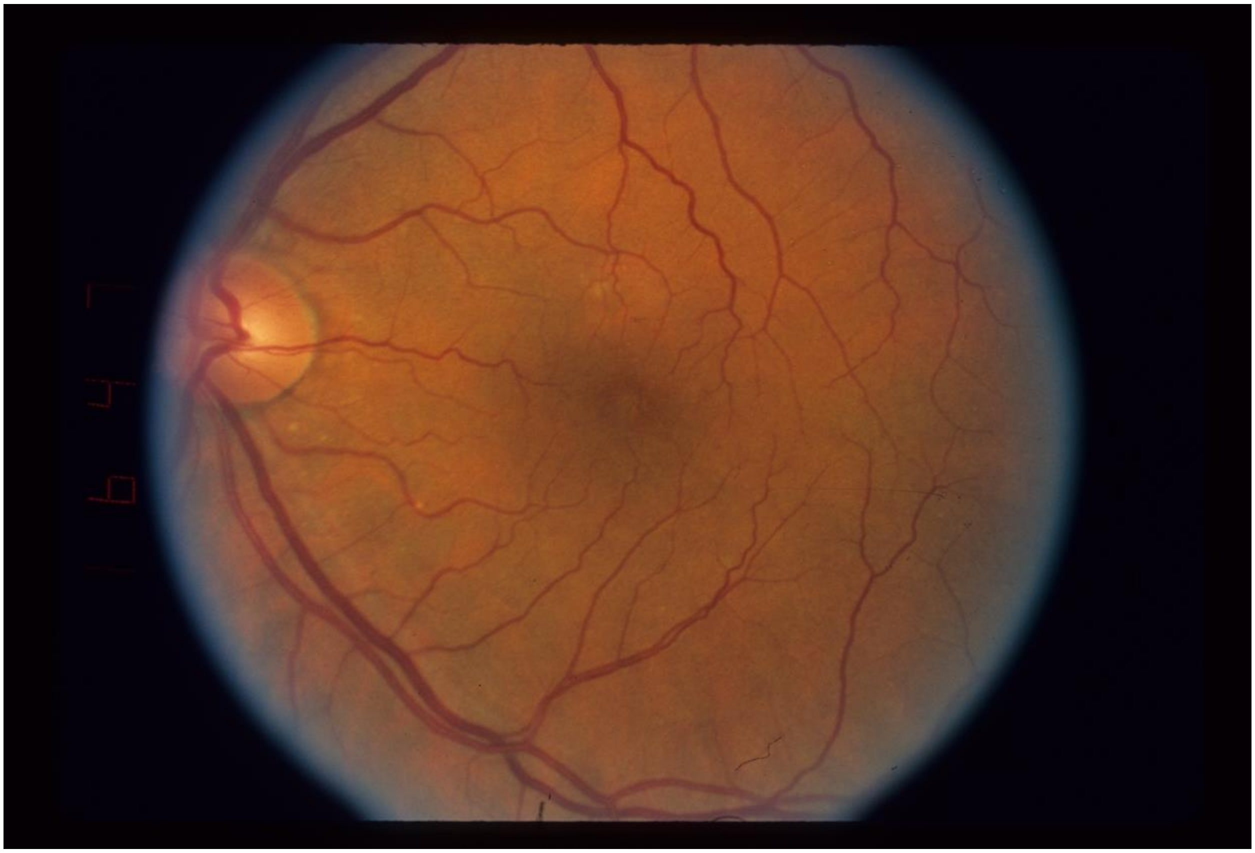

Figure S7: A fundus image from AREDS dataset.

Preprocessed:

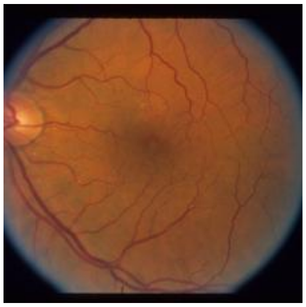

Figure S8: A preprocessed fundus image from AREDS dataset.

S3. GAN Model Details

Generator

U-Net [1] architecture is used for the generator because of its skip connections that enable it to pass low level structural information of its input to output easier than encoder-decoder architectures in which all the information should get passed through the bottleneck layer.

The number of channels in the generator's architecture (U-Net 256) [1, 2] is as follows:

$$3\ (input\ RGB\ image) \rightarrow 64 \rightarrow 128 \rightarrow 256 \rightarrow 512 \rightarrow 512 \rightarrow 512 \rightarrow 512 \rightarrow 512 \rightarrow 512 \rightarrow 1024 \rightarrow 1024 \rightarrow 1024 \rightarrow 1024 \rightarrow 512 \rightarrow 256 \rightarrow 128 \rightarrow 3\ (output\ RGB\ image)$$

Discriminator

It has been observed [2, 3] that using patch discriminator which penalizes its input's structure in the patch level rather than full image can still result in sharp realistic-looking (preserving high frequency information) generated images and also benefit from more computational efficiency because the size of patches can be smaller than the image. As mentioned in the paper, we used $70 * 70$ 'Patch' discriminator network. The number of channels in the discriminator's architecture is as follows:

$$6\ (3\ for\ first\ time\ point\ image + 3\ for\ predicted\ image\ of\ the\ generator) \rightarrow 64 \rightarrow 128 \rightarrow 256 \rightarrow 512 \rightarrow 1\ (discriminator\ scores\ for\ each\ patch)$$

Network Initialization

We followed Isola et al. [2] for network initialization, i.e., the generator and discriminator are initialized from a Gaussian distribution with zero mean and standard deviation equal to 0.02.

S4. Best Hyperparameter Setting for Classification Models

Table S2 summarizes hyperparameter settings which have achieved highest validation AUC for the experiments with the pairs having 2, 3, and 4 years gap. We tuned training batch size as well as Adam optimizer's [4] parameters: Learning Rate, β_1, β_2, and Weight Decay (coefficient of L2 regularization on the model's parameters).

Table S2: Description of the hyperparameter settings which achieved highest validation AUC in each experiment.

Model \ Parameter	Batch Size	Learning Rate	β_1	β_2	Weight Decay
2 years	128	0.0002	0.9	0.999	0.00001
3 years	32	0.0003	0.9	0.99	0.
4 years	128	0.0003	0.5	0.999	0.

S5. GAN Model's Longitudinal Predictions

Here, we provide more examples of our GAN model's longitudinal prediction for the cases who 1) have not been in advanced AMD condition in the baseline and did not progress to it until the end of study and 2) have been in advanced AMD in the baseline, and as there is not treatment for late AMD so far, have remained in advanced AMD until the end of study. In each figure, the left image in the first row is the baseline image, and the next ones are the predictions for the 4, 6, and 8 time points after baseline. The second rows contain their corresponding ground truth images.

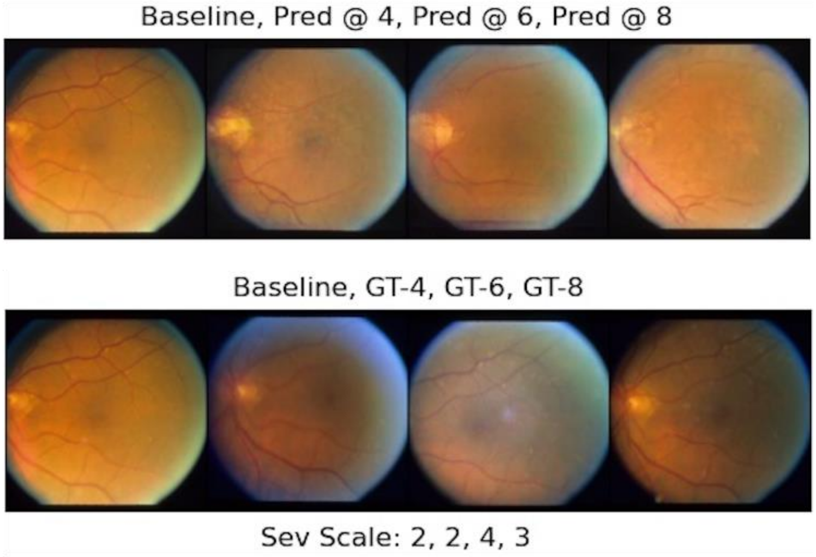

Figure S9: Longitudinal Prediction for the test image '52187_QUA_F2_LE_LS'

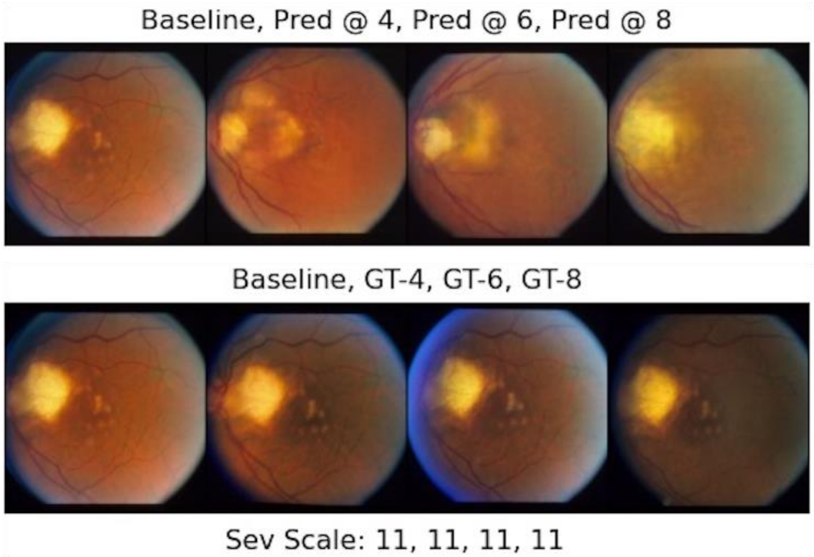

Figure S10: Longitudinal Prediction for the test image ‘52477_QUA_F2_LE_LS’

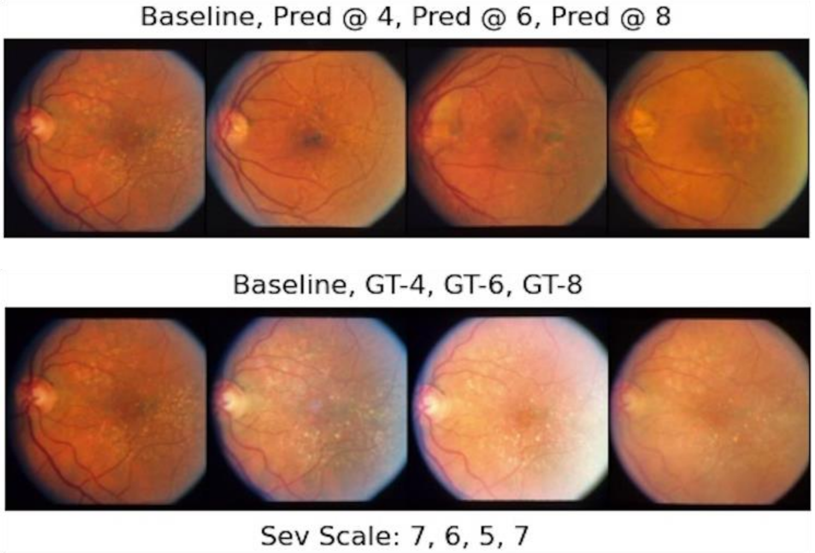

Figure S11: Longitudinal Prediction for the test image ‘52712_QUA_F2_LE_LS’

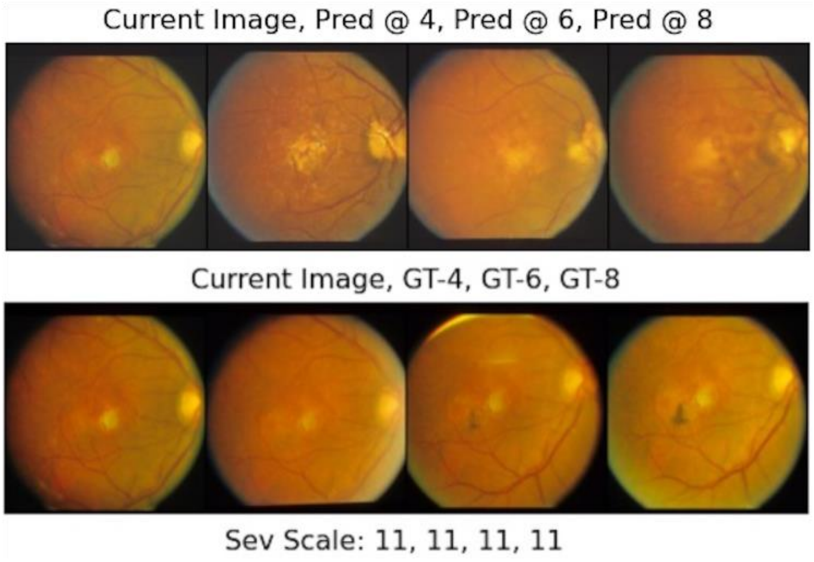

Figure S12: Longitudinal Prediction for the test image ‘53695_QUA_F2_RE_LS’

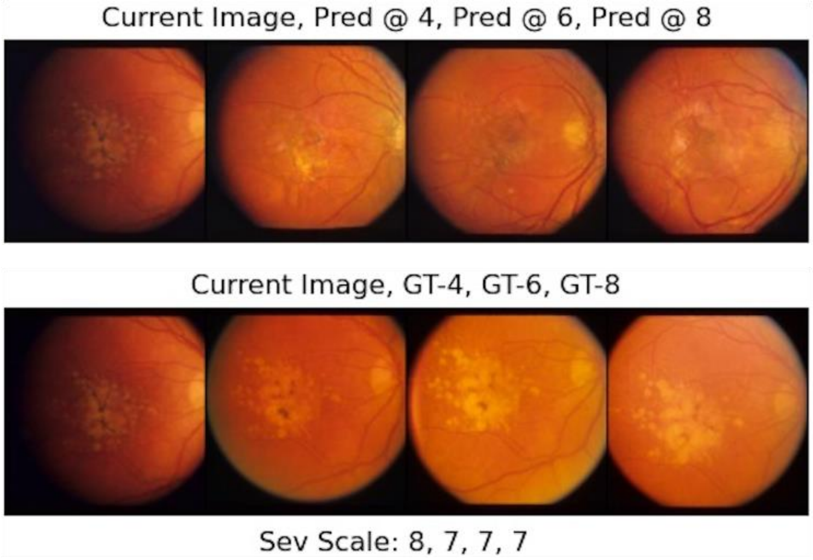

Figure S13: Longitudinal Prediction for the test image ‘55236_QUA_F2_RE_LS

S6. Images from UK Biobank used in our Experiments

Here, we list the name of images that we selected from UKBiobank to perform the validation experiments of our model on an independent dataset.

'2656719_21015_0_0.png', '4680067_21015_0_0.png', '5375555_21015_0_0.png',
'3244608_21015_0_0.png', '5672573_21015_0_0.png', '5471567_21015_0_0.png',
'2638597_21015_0_0.png', '1339583_21015_0_0.png', '4440932_21015_0_0.png',
'1154900_21015_0_0.png', '2744224_21015_0_0.png', '4588793_21015_0_0.png',

'5337019_21015_1_0.png',
'2209654_21015_0_0.png',
'1615790_21015_0_0.png',
'1999327_21015_1_0.png',
'5539112_21015_0_0.png',
'1370355_21015_0_0.png',
'3573517_21015_1_0.png',
'5107296_21015_0_0.png',
'1150836_21015_1_0.png',
'2351007_21015_1_0.png',
'2141048_21015_0_0.png',
'4174527_21015_0_0.png',
'4109760_21016_0_0.png',
'2213515_21016_1_0.png',
'1392869_21016_0_0.png',
'4405680_21016_1_0.png',
'2403821_21016_0_0.png',
'4440932_21016_1_0.png',
'2744224_21016_0_0.png',
'1606904_21016_0_0.png',
'3496199_21016_0_0.png',
'1121694_21016_0_0.png',
'3648579_21016_0_1.png',
'3573517_21016_1_0.png',
'5107296_21016_0_0.png',
'2325485_21016_0_0.png',
'3960358_21016_1_0.png',
'1484071_21016_1_0.png',
'4010491_21016_1_0.png',
'4136809_21016_1_0.png',
'1322758_21015_0_0.png',
'1010808_21015_0_0.png',
'1011176_21015_0_0.png',
'1008699_21015_0_0.png',
'1002470_21015_0_0.png',
'1008055_21015_0_0.png',
'2656470_21015_1_0.png',
'1016168_21015_0_0.png',
'1005010_21015_0_0.png',
'1008254_21015_0_0.png',
'1001589_21015_0_0.png',
'1008700_21015_0_0.png',
'1010218_21015_0_0.png',
'1008624_21015_0_0.png',
'5761504_21015_1_0.png',
'2035107_21015_0_0.png',
'1012763_21015_1_0.png',
'5287588_21015_1_0.png',
'4083652_21015_0_0.png',
'4273448_21015_1_0.png',
'1017387_21015_0_0.png',
'4405680_21015_1_0.png',
'4241459_21015_0_0.png',
'5304937_21015_0_0.png',
'4086631_21015_0_0.png',
'1284726_21015_0_0.png',
'4357916_21015_0_0.png',
'2629200_21015_1_0.png',
'4205712_21015_0_0.png',
'5970733_21015_1_0.png',
'1484071_21015_0_0.png',
'3902464_21015_0_0.png',
'5786357_21015_0_0.png',
'3100719_21016_1_0.png',
'1419085_21016_1_0.png',
'3100719_21016_0_0.png',
'2209654_21016_0_0.png',
'4941659_21016_1_0.png',
'1533640_21016_0_0.png',
'3837454_21016_0_0.png',
'4588793_21016_0_0.png',
'4338330_21016_0_0.png',
'3990923_21016_0_0.png',
'4174527_21016_0_0.png',
'4249998_21016_0_0.png',
'1750895_21016_0_0.png',
'1284726_21016_0_0.png',
'3699182_21016_0_0.png',
'3134276_21016_0_0.png',
'2395147_21016_0_0.png',
'1013328_21015_0_0.png',
'1006763_21015_0_0.png',
'1017731_21015_0_0.png',
'1444330_21015_1_0.png',
'1020547_21015_0_0.png',
'3965339_21015_0_0.png',
'5441276_21015_1_0.png',
'1004811_21015_0_0.png',
'3984587_21015_1_0.png',
'4316008_21015_1_0.png',
'1002488_21015_0_0.png',
'1009124_21015_0_0.png',
'1013237_21015_0_0.png',
'1926576_21015_1_0.png',
'1004923_21015_0_0.png',
'1018880_21015_0_0.png',
'5663680_21015_0_0.png',
'5266648_21015_0_0.png',
'1003574_21015_0_0.png',
'1002520_21015_0_0.png',
'1009257_21015_0_0.png',
'3950059_21015_1_0.png',
'1750895_21015_0_0.png',
'1484071_21015_1_0.png',
'1643086_21015_0_0.png',
'2478715_21015_0_0.png',
'4246134_21015_0_0.png',
'5266648_21015_1_0.png',
'5258276_21015_0_0.png',
'5238716_21015_1_0.png',
'1161726_21015_0_0.png',
'5383962_21015_1_0.png',
'1492593_21015_0_0.png',
'4088926_21016_0_0.png',
'3807453_21016_0_0.png',
'3341807_21016_0_0.png',
'3414644_21016_0_0.png',
'5471567_21016_0_0.png',
'1615790_21016_0_0.png',
'2430547_21016_0_0.png',
'3292112_21016_0_0.png',
'3442579_21016_1_0.png',
'4440932_21016_0_0.png',
'3483443_21016_0_0.png',
'2469100_21016_1_0.png',
'5266648_21016_1_0.png',
'2629200_21016_1_0.png',
'4947990_21016_1_0.png',
'3476804_21016_1_0.png',
'2667605_21016_0_0.png',
'4253423_21016_1_0.png',
'5502654_21015_1_0.png',
'1008139_21015_0_0.png',
'1014276_21015_0_0.png',
'1012339_21015_1_0.png',
'4755734_21015_1_0.png',
'1015838_21015_0_0.png',
'1001614_21015_0_0.png',
'1548759_21015_0_0.png',
'1010606_21015_1_0.png',
'4200584_21015_1_0.png',
'1011111_21015_0_0.png',
'2071571_21015_0_0.png',
'1000777_21015_0_0.png',
'1001030_21015_0_0.png',
'1011497_21015_1_0.png',
'3260074_21015_1_0.png',
'2477330_21015_1_0.png',
'1013730_21015_0_0.png',
'1011991_21015_1_0.png',
'3642045_21015_1_0.png',
'1009640_21015_0_0.png',
'2103598_21015_1_0.png',

'3652494_21015_1_0.png',
'1003400_21015_1_0.png',
'1010205_21015_0_0.png',
'3437565_21015_0_0.png',
'1007733_21015_0_0.png',
'1009146_21015_1_0.png',
'2106665_21015_0_0.png',
'1011963_21015_0_0.png',
'5988797_21015_1_0.png',
'1007460_21015_0_0.png',
'1012951_21015_0_0.png',
'5354834_21015_1_0.png',
'4005760_21016_1_0.png',
'1003338_21016_1_0.png',
'2656470_21016_1_0.png',
'1008699_21016_0_0.png',
'5607521_21016_1_0.png',
'4583685_21016_1_0.png',
'4151986_21016_1_0.png',
'4755734_21016_1_0.png',
'3327696_21016_1_0.png',
'1001030_21016_0_0.png',
'1001739_21016_1_0.png',
'2035107_21016_0_0.png',
'4010442_21016_0_0.png',
'5988797_21016_1_0.png',
'1222783_21016_1_0.png',
'1003743_21016_0_0.png',
'1010223_21016_0_0.png',
'1010770_21016_1_0.png',
'3804862_21016_1_0.png',
'2841504_21016_0_0.png',
'1009426_21016_0_0.png',
'1003909_21016_0_0.png',
'1000853_21016_0_0.png',
'3772387_21016_1_0.png',
'1827183_21016_1_0.png',
'1011361_21016_0_0.png',
'1008055_21016_0_0.png',
'1010606_21016_1_0.png',
'5880878_21016_1_0.png',
'5502654_21016_1_0.png',
'1322758_21016_0_0.png',
'1001589_21016_0_0.png',
'2632220_21016_1_0.png',
'1016131_21015_0_0.png',
'1003472_21015_0_0.png',
'1002971_21015_0_0.png',
'1007583_21015_1_0.png',
'1016515_21015_0_0.png',
'2632220_21015_1_0.png',
'1000214_21015_0_0.png',
'1015464_21015_0_0.png',
'1008683_21015_0_0.png',
'3882609_21015_1_0.png',
'2841504_21015_0_0.png',
'1007390_21015_0_0.png',
'3426452_21016_1_0.png',
'4723254_21016_1_0.png',
'1010770_21016_0_0.png',
'4513232_21016_1_0.png',
'1002758_21016_1_0.png',
'1004811_21016_0_0.png',
'4592210_21016_1_0.png',
'1006734_21016_0_0.png',
'3422593_21016_1_0.png',
'1008139_21016_0_0.png',
'1009925_21016_0_0.png',
'3260074_21016_0_0.png',
'1005010_21016_0_0.png',
'1002488_21016_0_0.png',
'1000827_21016_0_0.png',
'1010487_21016_0_0.png',
'5493124_21016_1_0.png',
'1010205_21016_0_0.png',
'1007733_21016_0_0.png',
'4611536_21016_1_0.png',
'1009008_21016_0_0.png',
'1002192_21016_0_0.png',
'1004515_21016_0_0.png',
'3965339_21016_0_0.png',
'1005826_21016_0_0.png',
'1008700_21016_0_0.png',
'1004775_21016_0_0.png',
'2017481_21016_1_0.png',
'1098399_21016_1_0.png',
'2438381_21016_0_0.png',
'5958517_21016_1_0.png',
'1009124_21016_0_0.png',
'5354834_21016_1_0.png',
'5493124_21015_1_0.png',
'1018061_21015_0_0.png',
'2438381_21015_1_0.png',
'1006473_21015_0_0.png',
'1009665_21015_0_0.png',
'5720879_21015_1_0.png',
'2017481_21015_1_0.png',
'1699477_21015_0_0.png',
'4592210_21015_1_0.png',
'4151986_21015_1_0.png',
'1016308_21015_0_0.png',
'1006763_21016_0_0.png',
'2071571_21016_0_0.png',
'1922955_21016_1_0.png',
'5354929_21016_1_0.png',
'1007445_21016_0_0.png',
'1006473_21016_0_0.png',
'5287588_21016_1_0.png',
'3984587_21016_1_0.png',
'1007445_21016_1_0.png',
'1890556_21016_0_0.png',
'1444330_21016_1_0.png',
'1010163_21016_0_0.png',
'1000295_21016_0_0.png',
'1010668_21016_1_0.png',
'1008772_21016_0_0.png',
'5266648_21016_0_0.png',
'4136809_21016_0_0.png',
'1567613_21016_1_0.png',
'1041877_21016_1_0.png',
'5720879_21016_1_0.png',
'1011176_21016_0_0.png',
'1001247_21016_1_0.png',
'1011472_21016_0_0.png',
'1000509_21016_0_0.png',
'4200584_21016_1_0.png',
'5761504_21016_1_0.png',
'1007390_21016_0_0.png',
'3396054_21016_1_0.png',
'1000649_21016_1_0.png',
'1010808_21016_0_0.png',
'1000777_21016_0_0.png',
'1000425_21016_0_0.png',
'1007460_21016_0_0.png',
'3404359_21016_1_0.png'

S7. Model's Prediction Results on UK Biobank Dataset

Table S3 provides statistics regarding the performance of our checkpoint models trained on AREDS dataset on the independent test dataset containing 300 images selected from UK Biobank dataset [5].

Table S3: Accuracy and Confusion Matrix of the models trained with the pairs with 2(A), 3(B), and 4(C) years gap between successive time points on the independent validation set chosen from UK Biobank dataset. In the confusion matrices, label '0' is not being in advanced AMD and '1' vice versa.

Model	Accuracy	Confusion Matrix
A	0.8667	$\begin{bmatrix} 197 & 3 \\ 37 & 63 \end{bmatrix}$
B	0.9233	$\begin{bmatrix} 191 & 9 \\ 14 & 86 \end{bmatrix}$
C	0.9267	$\begin{bmatrix} 188 & 12 \\ 10 & 90 \end{bmatrix}$

S8. Structure of Adding Age Information to the Inputs of the Classifier

To explore whether explicitly providing the age information to the model can improve the performance we concatenate age value to the representations of the outputs of the global average pooling layer in ResNet-18 [6] architecture. The procedure is shown in Figure S6.

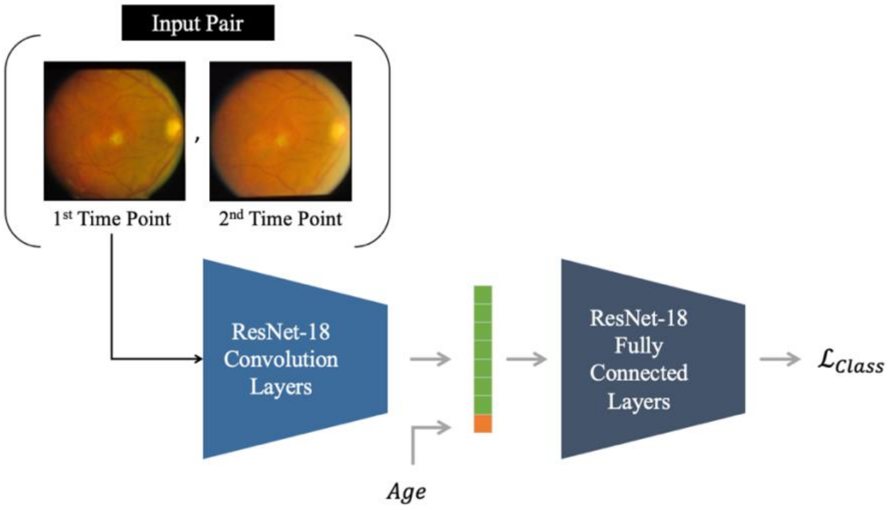

Figure S14: The classifier architecture when using both fundus image and age information as its inputs.

S9. Classification Model's Results on Grading the Fundus Images

We compare our model's performance on the task of grading its input image with the late AMD grading module in DeepSeeNet [7].

Table S4: Our model's classifier statistics for the task of grading the first time point image (advanced/not advanced AMD) in the pairs. We use the confusion matrix predictions of the model on the test set and combine classes y ϵ {0,1} as both of these classes correspond to the first time point (input) not being in advanced AMD.

T (Time Gap in Units of 6 Months)	Binarized Confusion Matrix	Accuracy	Sensitivity	Specificity
4 (2 years)	$\begin{bmatrix} 503 & 20 \\ 66 & 295 \end{bmatrix}$	0.902	0.817	0.962
6 (3 years)	$\begin{bmatrix} 466 & 28 \\ 51 & 232 \end{bmatrix}$	0.898	0.819	0.943
8 (4 years)	$\begin{bmatrix} 444 & 35 \\ 26 & 213 \end{bmatrix}$	0.915	0.891	0.926

References

1. Ronneberger, O., P. Fischer, and T. Brox. *U-Net: Convolutional Networks for Biomedical Image Segmentation*. 2015. Cham: Springer International Publishing.
2. Isola, P., et al. *Image-to-image translation with conditional adversarial networks*. in *Proceedings of the IEEE conference on computer vision and pattern recognition*. 2017.
3. Li, C. and M. Wand. *Precomputed real-time texture synthesis with markovian generative adversarial networks*. in *European conference on computer vision*. 2016. Springer.
4. Kingma, D.P. and J. Ba, *Adam: A method for stochastic optimization.* arXiv preprint arXiv:1412.6980, 2014.
5. Sudlow, C., et al., *UK biobank: an open access resource for identifying the causes of a wide range of complex diseases of middle and old age.* Plos med, 2015. **12**(3): p. e1001779.
6. He, K., et al. *Deep residual learning for image recognition*. in *Proceedings of the IEEE conference on computer vision and pattern recognition*. 2016.
7. Peng, Y., et al., *DeepSeeNet: A Deep Learning Model for Automated Classification of Patient-based Age-related Macular Degeneration Severity from Color Fundus Photographs.* Ophthalmology, 2019. **126**(4): p. 565-575.
